# Topoisomerase 1 prevents replication stress at R-loop-enriched transcription termination sites

**DOI:** 10.1038/s41467-020-17858-2

**Published:** 2020-08-07

**Authors:** Alexy Promonet, Ismaël Padioleau, Yaqun Liu, Lionel Sanz, Anna Biernacka, Anne-Lyne Schmitz, Magdalena Skrzypczak, Amélie Sarrazin, Clément Mettling, Maga Rowicka, Krzysztof Ginalski, Frédéric Chedin, Chun-Long Chen, Yea-Lih Lin, Philippe Pasero

**Affiliations:** 1 Institut de Génétique Humaine, CNRS et Université de Montpellier, Equipe labélisée Ligue contre le Cancer, Montpellier, France; 2 Institut Curie, PSL Research University, CNRS, UMR3244, Sorbonne Université, Paris, France; 3 Department of Molecular and Cellular Biology, University of California, Davis, CA 95616 USA; 4 Laboratory of Bioinformatics and Systems Biology, Centre of New Technologies, University of Warsaw, Warsaw, Poland; 5 BioCampus Montpellier, CNRS et Université de Montpellier, Montpellier, France; 6 Institut de Génétique Humaine, CNRS et Université de Montpellier, Montpellier, France; 7 Department of Biochemistry and Molecular Biology, University of Texas Medical Branch at Galveston, Galveston, TX USA; 8 Present Address: Institut Gustave Roussy, Villejuif, France

**Keywords:** Chromosomes, Genetics

## Abstract

R-loops have both positive and negative impacts on chromosome functions. To identify toxic R-loops in the human genome, here, we map RNA:DNA hybrids, replication stress markers and DNA double-strand breaks (DSBs) in cells depleted for Topoisomerase I (Top1), an enzyme that relaxes DNA supercoiling and prevents R-loop formation. RNA:DNA hybrids are found at both promoters (TSS) and terminators (TTS) of highly expressed genes. In contrast, the phosphorylation of RPA by ATR is only detected at TTS, which are preferentially replicated in a head-on orientation relative to the direction of transcription. In Top1-depleted cells, DSBs also accumulate at TTS, leading to persistent checkpoint activation, spreading of γ-H2AX on chromatin and global replication fork slowdown. These data indicate that fork pausing at the TTS of highly expressed genes containing R-loops prevents head-on conflicts between replication and transcription and maintains genome integrity in a Top1-dependent manner.

## Abstract (web summary)

While R-loops can alter cell homeostasis, it is unclear what determines their toxicity. Here, the authors, by using Top1 knockdown as a tool to enhance the formation of R-loops at certain genomic sites, reveal and characterize a proportion of R-loops that are more toxic to the cell by causing DNA damage.

## Introduction

Replication stress (RS) refers to a variety of events of endogenous or exogenous origin that interfere with the progression of replication forks^1^. In precancerous lesions, RS is induced by deregulated oncogenes and promotes cancer development by increasing genomic instability^2^. RS occurs spontaneously at specific regions of the genome that are intrinsically difficult to replicate, such as secondary DNA structures, DNA lesions, highly expressed genes, or chromatin-bound protein complexes^1^. The mechanism by which these obstacles interfere with fork progression and promote genomic instability remains poorly understood.

DNA synthesis initiates at thousands of replication origins distributed throughout the genome^3^. Replication forks progress bidirectionally from these origins and activate the ATR-CHK1 pathway when they encounter obstacles. This surveillance mechanism detects single-stranded DNA (ssDNA) accumulating at stalled forks. Checkpoint signaling is initiated with the binding of the ATR kinase and its partner ATRIP to the ssDNA-binding complex RPA^4^. Once activated by TopBP1, ATR phosphorylates multiple targets, including the RPA32 subunit on S33 (called thereafter p-RPA) and the histone variant H2AX on S139 (γ-H2AX). Unlike RPA, which concentrates at stalled forks, H2AX can also be phosphorylated by ATM and spreads over megabases of DNA in response to DNA double-strand breaks (DSBs)^1^. ATR activates the effector kinase CHK1 to amplify the checkpoint response, repress late replication origins and prevent premature entry into mitosis^5,6^.

The transcription and replication machineries share the same DNA template, which renders head-on (HO) or codirectional (CD) collisions difficult to avoid^7,8^. As HO collisions are more deleterious than CD collisions, eukaryotic and prokaryotic cells have evolved strategies to prevent HO conflicts^9–13^, including a bias for the most transcribed genes towards a CD orientation with the direction of replication forks^8,14–16^. However, the molecular consequences of frontal collisions between replication and transcription have remained largely unexplored at the genome-wide level in human cells.

Besides HO collisions, transcription–replication conflicts (TRCs) can also be caused by three-stranded nucleic acids structures called R-loops, which contain a RNA:DNA hybrid and a displaced DNA strand^17^. R-loops are formed co-transcriptionally when the nascent RNA reanneals with the template DNA strand, leaving the non-coding strand unpaired^18,19^. They assemble at specific sites determined both by DNA sequence and topological state^20,21^. Genome-wide analyses indicate that they are abundant in the human genome, covering up to 5% of unique sequences^22^. R-loops assemble dynamically at transcription initiation and termination sites, where they contribute to the regulation of gene expression and to transcription termination^19,23–25^. They are also involved in processes, such as class-switch recombination of immunoglobulin genes^26^, chromatin patterning^18^, and telomere maintenance^27^. In addition to this growing number of physiological functions, studies in model organisms have shown that R-loops have deleterious effects by increasing genomic instability^28^. However, the mechanisms by which R-loops induce RS in human cells remain to be clarified.

R-loop homeostasis is regulated by pathways controlling their formation or their degradation^29^. R-loop assembly increases in the absence of factors involved in the maturation or the export of mRNAs, such as the THO/TREX complex^30^. It is also facilitated by negative DNA supercoiling, which is normally relaxed by topoisomerase I (Top1)^17,31^. Once formed, RNA:DNA hybrids can be degraded by RNase H or resolved by specific helicases^10,18,19^. Alterations of R-loop metabolism promote RS and genomic instability^10,32,33^. As RS in these mutants is generally relieved by the ectopic expression of RNase H, it has been proposed that R-loops physically interfere with the progression of replication forks^11,34–36^. Yet, it is still unclear whether all R-loops are equally harmful to forks or whether specific structural or contextual features determine their toxicity. To address these questions, we have mapped RNA:DNA hybrids in the human genome and compared their distribution relative to replication stress markers and DSBs in control and Top1-depleted HeLa cells. We show that although marks of spontaneous replication stress are mostly found at R-loop containing loci, most of the R-loops are not associated with replication stress markers. We also show that RPA is phosphorylated by ATR at the TTS of genes that are replicated in a head-on orientation relative to the direction of transcription and that DSBs form at these sites in Top1-depleted cells. These data suggest that interference between replication and transcription occurs preferentially at TTS and that Top1 helps resolve these conflicts by preventing the formation of R-loops and by relaxing DNA supercoiling.

## Results

### Top1 depletion increases R-loop levels

**Fig. 1:**
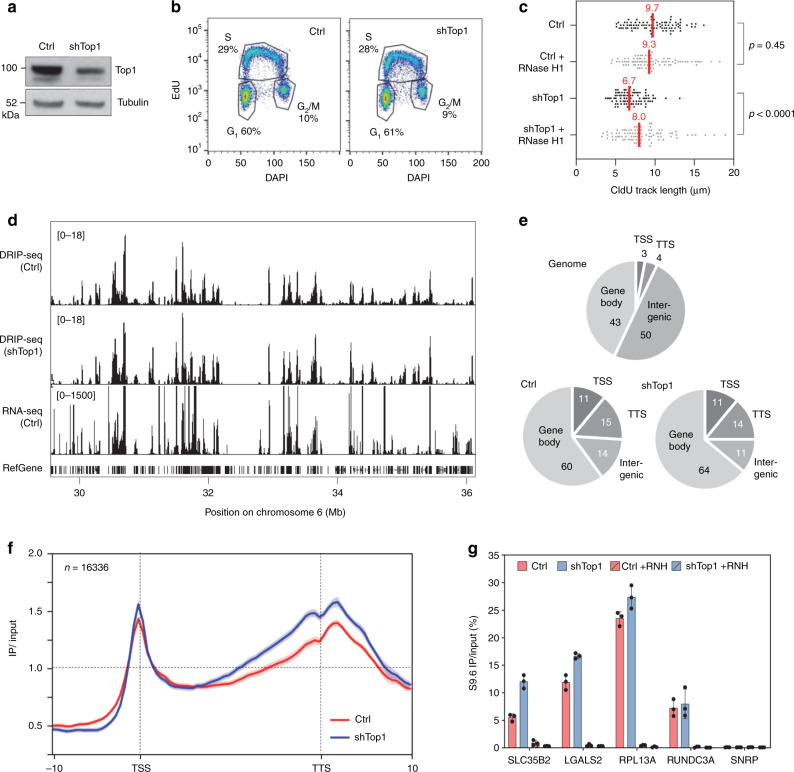
Depletion of Top1 increases R-loop formation and slows down fork progression. **a** Western blot analysis of Top1 levels in HeLa cells expressing shRNA targeting Top1 (shTop1) under the control of a doxycycline-inducible promoter at 72 h post-induction (*n* = 9). **b** Cell-cycle distribution of control and shTop1 cells determined by flow cytometry after labeling of S-phase cells with EdU. The fraction of cells in the different cell-cycle phases is indicated. See Supplementary Fig. 7 for gating strategy. **c** Doxycycline-treated control and shTop1 HeLa cells were transfected for 48 h with a mock vector (EGFP-N1) or human RNase H1-EGFP (+RNase H1) and were sequentially labeled with IdU and CldU for 20 min. Replication fork progression was measured using DNA fiber spreading as described in “Methods” section. The median length of CldU tracks is indicated in red. At least 150 fibers of each sample were measured (*n* = 3). *P*-values were calculated with the two-sided Mann–Whitney rank-sum test. **d** DRIP-seq analysis of the distribution of RNA:DNA hybrids expressed in RPKM (Read Per Kilobase per Million reads) in control and shTop1 HeLa cells. A representative region on chromosome 6 is shown. RNA-seq data (RPKM) for HeLa cells and gene positions (hg19) are also indicated. **e** Distribution of R-loop peaks relative to gene annotations in control and shTop1 HeLa cells. Peaks were obtained with MACS2 and were analyzed with CEAS (Cis-Regulatory Element Annotation System). The expected distribution in case peaks were randomly positioned in the genome is shown for comparison. The percentage of DRIP-seq signals present in each annotation class is indicated. TSS: Transcription Start Site (5′-UTR and 3 kb upstream). TTS: Transcription Termination Site (3′-UTR and 3 kb downstream). **f** Metaplot of the distribution of S9.6 signals (IP/input) along 16,336 active human genes (RPKM > 0) and flanking regions (±10 kb) in control (red) and shTop1 (blue) HeLa cells. Error bars correspond to SEM. **g** DRIP-qPCR analysis of the relative enrichment of RNA:DNA hybrids at the TTS of four genes and a negative control regions (SNPRN) in control and shTop1 HeLa cells after RNase H1 treatment (+RNH). Error bars correspond to three independent experiments.

Top1 is essential for cell growth and an acute depletion of this enzyme leads to a G_0_/G_1_ arrest^37^. To monitor the effect of Top1 depletion on TRCs, we constructed a stable HeLa cell line expressing an inducible shRNA against Top1 (shTop1). Conditions of depletion were optimized to reduce Top1 levels without altering cell-cycle progression. This is confirmed by the fact that the distribution of cells in G_1_, S, and G_2_/M phases of the cell cycle was not affected by the reduction of Top1 levels (Fig. 1a, b). To monitor the impact of this depletion on replication forks, cells were labeled for 20 min with 5-iodo-2′-deoxyuridine (IdU) and for 20 min with 5-chloro-2′-deoxyuridine (CldU). DNA fibers were spread on glass slides and the incorporation of halogenated thymidine analogs was detected by immunofluorescence using specific antibodies^38^. We observed a 30–40% reduction of CldU tracks length in Top1-depleted cells relative to control cells, which was largely suppressed by a transient overexpression of human RNase H1 (Fig. 1c). These data are consistent with our earlier study^35^ and suggest that the replication slowdown observed in shTop1 cells is caused by RNA:DNA hybrids. To confirm that Top1-depleted cells have increased levels of R-loops, we used the S9.6 monoclonal antibody^39^ to quantify RNA:DNA hybrids in control and shTop1 cells by slot blot (Supplementary Fig. 1a). We observed a 70% increase in R-loop levels in shTop1 cells, which is consistent with the increase recently reported in human HEK293 cells transfected with a pool of Top1 siRNAs^37^. Importantly, this signal was highly sensitive to RNase H (Supplementary Fig. 1a, b), indicating that it corresponds to RNA:DNA hybrids.

### R-loops form preferentially at TSS and TTS

To identify regions of the human genome that are prone to form R-loops in the absence of Top1, RNA:DNA hybrids were immunoprecipitated with the S9.6 antibody and were analyzed by next-generation sequencing (DRIP-seq) as described earlier^23^. DRIP-seq profiles revealed the presence of discrete peaks (Fig. 1d) that overlapped with 8726 and 10,906 annotated genes (RefSeq annotations, hg19) in control and shTop1 cells, respectively (Supplementary Fig. 1c). Most of R-loop-positive genes (8015) were common to both cell types. On average, genes with DRIP-seq signal were more transcribed than others (Supplementary Fig. 1d) and were enriched in RNA:DNA hybrids at both transcription start sites (TSS) and transcription termination sites (TTS; Fig. 1e, f), which is consistent with earlier studies^22^. The enrichment of R-loops at the TTS of a subset of genes was confirmed by DRIP-qPCR (Fig. 1g). Genes enriched in R-loops in the absence of Top1 showed a similar distribution of DRIP signal and a similar level of expression relative to control cells (Supplementary Fig. 1e, f). In shTop1 cells, DRIP signals were further increased at the TTS of converging genes in a manner that depended both on the distance between converging genes (Supplementary Fig. 1g) and on their level of expression (Supplementary Fig. 1h). Interestingly, R-loop containing genes that were specific to shTop1 cells showed lower mRNA levels relative to R-loop enriched genes common to both cell types (Supplementary Fig. 1i) and showed higher DRIP levels than R-loop containing genes that are specific to control cells (Supplementary Fig. 1j). Together, these data indicate that the TSS and TTS of highly expressed genes represent hotspots of R-loops and that shTop1 cells show increased R-loop levels and slower fork progression.

### Phospho-RPA accumulates at TTS of R-loop containing genes

**Fig. 2:**
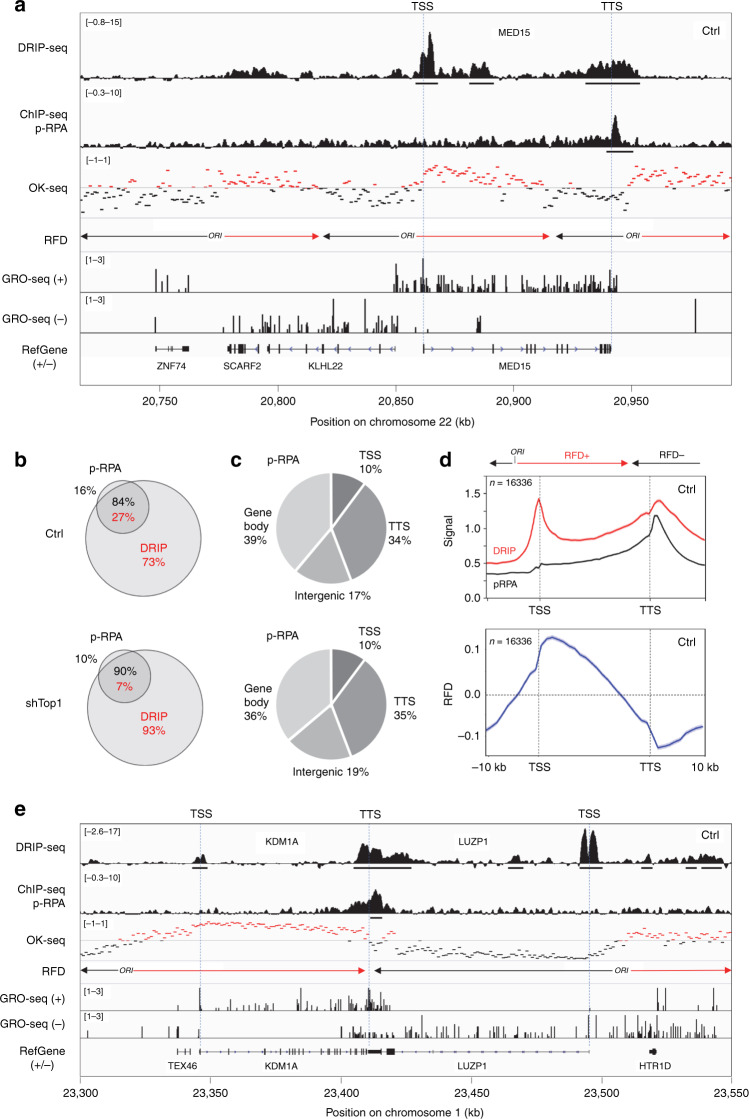
Phospho-RPA accumulates at TTS in control and shTop1 cells. **a** Distribution of RNA:DNA hybrids (DRIP-seq), p-RPA32 S33 (ChIP-seq), Okazaki fragments (OK-seq), and nascent transcription (GRO-seq) signals at a representative region on chromosome 22 in control HeLa cells. Replication fork direction (RFD) is derived from OK-seq data. The positions of TSS and TTS are indicated for the MED15 gene. The positions of DRIP and p-RPA peaks identified with MACS2 are also indicated. **b** Venn diagram of the percentage of genes overlapping with R-loop (red) and p-RPA peaks (black) peaks (MACS2) in control and shTop1 cells. **c** The distribution of p-RPA peaks was analyzed with CEAS as in Fig. 1e. The percentage of p-RPA peaks present in each region is indicated. **d** Metaplots of RNA:DNA hybrids (DRIP, red), p-RPA (black), and replication fork direction (RFD, blue) at 16,336 active genes in HeLa cells. Error bars indicate SEM. **e** Distribution of RNA:DNA hybrids (DRIP-seq), p-RPA32 S33 (ChIP-seq), Okazaki fragments (OK-seq), and nascent transcription (GRO-seq) signals at two converging genes on chromosome 1 in control HeLa cells. The positions of DRIP and p-RPA peaks identified with MACS2 are indicated.

To identify RNA:DNA hybrids that may interfere with fork progression, we next used the phosphorylation of RPA32 by ATR on S33 (p-RPA) as a surrogate for stalled replication and ATR activation. Regions enriched in p-RPA were mapped by ChIP-seq and were positioned relative to DRIP signals in untreated control cells (Fig. 2a) and in shTop1 cells (Supplementary Fig. 2a, b). Levels of p-RPA were determined on chromatin by western blot (Supplementary Fig. 2c) and at specific loci by ChIP-qPCR (Supplementary Fig. 2d). Both assays revealed an increase in p-RPA levels in shTop1 cells, which is consistent with their increased replication stress relative to control cells, as illustrated by DNA fiber analysis (Fig. 1c). However, the analysis of ChIP-seq profiles revealed that 97% of the p-RPA S33 sites observed in shTop1 cells were also detected in control cells (Supplementary Fig. 2a, b), indicating that the same sites accumulate p-RPA after Top1 knockdown. Moreover, the comparison of the position of individual DRIP and p-RPA peaks (Fig. 2a; underlined) revealed that although most genes enriched in p-RPA contained R-loops, only a fraction of R-loop containing genes were enriched in p-RPA in control and shTop1 cells (Fig. 2b). Together, these data indicate that most R-loops do not interfere with fork progression.

To identify R-loops that are potentially toxic for replication forks, we compared the distribution of DRIP signals and p-RPA at annotated genes. Unlike R-loops, p-RPA was mostly present at TTS and not at TSS in control and shTop1 cells (Fig. 2c, d; Supplementary Fig. 2e). This is illustrated with the MED15 gene, which shows a peak of p-RPA downstream of TTS and no enrichment at TSS (Fig. 2a; Supplementary Fig. 2a). These data suggest that forks preferentially pause at the TTS of highly expressed genes containing R-loops.

### Phospho-RPA accumulates at TTS in a head-on orientation

HO collisions between replication and transcription are generally considered more harmful than CD collisions^9–11^. Since highly expressed genes usually contain active replication origins in their promoter region and are therefore mostly replicated codirectionally with transcription^14,15^, we reasoned that the asymmetric distribution of p-RPA at genes could reflect this bias in replication fork direction (RFD). To address this possibility, we analyzed the direction of fork movement at gene loci using Okazaki fragment sequencing data^14^. As illustrated in Fig. 2a, the MED15 gene contains a replication origin in its promoter region and is mostly replicated by forks progressing codirectionally with transcription. In contrast, the TTS region of MED15 is preferentially replicated by an origin located downstream of the gene. Remarkably, p-RPA enrichment was detected at TTS, where replication and transcription converge, and not at TSS, which is replicated in a CD orientation. This p-RPA enrichment at TTS regions replicated in a HO orientation (negative RFD), but not at TSS replicated in a CD orientation (positive RFD) was also observed on a metaplot of 16,336 active genes (Fig. 2d; Supplementary Fig. 2e), indicating that it is a general feature of the human genome.

As the TTS of converging genes are hotspots for RNA:DNA hybrids (Supplementary Fig. 1g, h), we next asked whether it is also the case for p-RPA. As illustrated in Fig. 2e and Supplementary Fig. 2f, p-RPA was enriched at the TTS of the converging genes KDM1A and LUZP1 in both control and shTop1 cells. Phospho-RPA was also enriched at the TTS of 2118 converging genes separated by <5 kb, but not for 3974 TTS separated by more than 5 kb (Supplementary Fig. 2g). The amount of p-RPA depended on the level of expression of converging genes (Supplementary Fig. 2h), as it is the case for R-loops (Supplementary Fig. 1h). Interestingly, p-RPA enrichment at TTS was also influenced by the presence of a nearby replication origin downstream of the TTS (Supplementary Fig. 2i), similar to what was observed for the MED15 gene (Fig. 2a). The amount of p-RPA at TTS decreased as the distance between TTS and the replication origin increased (Supplementary Fig. 2i), presumably because a short distance to the next downstream origin increases the risk of HO collisions at TTS (Supplementary Fig. 2j; negative RFD). Altogether, these data indicate that the accumulation of p-RPA at TTS is determined by the direction of replication forks and gene transcription.

### Top1-depleted cells accumulate γ-H2AX and DSBs

**Fig. 3:**
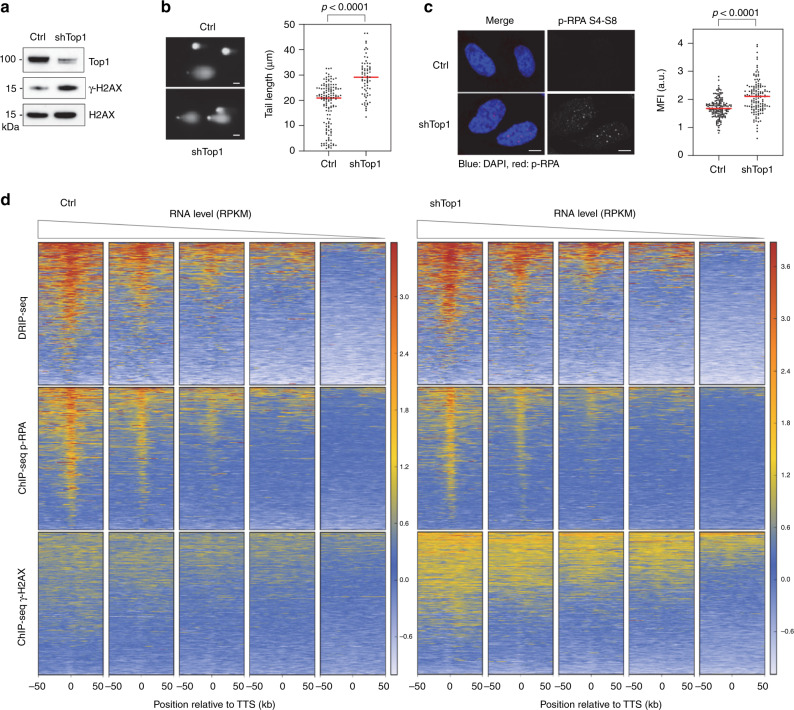
Top1 prevents the accumulation of γ-H2AX at highly expressed genes. **a** Western blot analysis of γ-H2AX levels in control and shTop1 cells (*n* = 3). **b** Analysis of DNA breaks in control and shTop1 cells. Representative images and the distribution of comet tail lengths are shown. Median of tail length is indicated in red. At least 50 cells of each sample were measured (*n* = 2). Bar is 10 µm. *P*-values were calculated with the two-sided Mann–Whitney rank-sum test. **c** Immunodetection of phospho-RPA32 S4/S8 in control and shTop1 cells. Mean fluorescence intensity (MFI) of the p-RPA32 S4/S8 signals is indicated in red. At least 400 cells of each sample were quantified (*n* = 3). *P*-values were calculated with the two-sided Mann–Whitney rank-sum test. Bar is 5 μm. **d** Heat map of the intensity of RNA:DNA hybrids (DRIP), p-RPA and γ-H2AX at TTS in control and shTop1 HeLa cells for five groups of genes with decreasing expression levels (RNA-seq). In each group, genes were sorted relative to the intensity of DRIP signal.

To further characterize the impact of R-loops on replication stress and chromosome breaks, we next analyzed the presence of γ-H2AX in control and shTop1 cells. Western blot analyses revealed a global increase of γ-H2AX levels in shTop1 cells relative to control cells (Fig. 3a). This is consistent with an increase of spontaneous DNA breaks in shTop1 cells relative to control cells, as determined by comet assay (Fig. 3b) and to an increase of p-RPA32 S4/S8 foci in shTop1 cells, which is indicative of DSBs^40^ (Fig. 3c). To determine whether DSBs accumulate at the TTS of highly expressed genes containing R-loops, we next analyzed the distribution of γ-H2AX by ChIP-seq. As a positive control, we immunoprecipitated γ-H2AX in the DIvA (DSB inducible via *Asi*SI) human cell line^41^. As expected, we observed a spreading of the γ-H2AX signal over megabases from *Asi*SI sites after DSB induction (+Tam), but not in untreated cells (−Tam; Supplementary Fig. 3a). To analyze the distribution of γ-H2AX around TTS in control and shTop1 HeLa cells, the 35,251 annotated genes were sorted according to their mRNA level (RPKM) and were organized in five quintiles (7050 genes) of decreasing gene expression level (Fig. 3d). Remarkably, γ-H2AX was mostly detected at the TTS of highly expressed genes and only in shTop1 cells. This difference contrasts with the distribution of R-loop and p-RPA signals, which were comparable in both cell types (Fig. 3d). The γ-H2AX signal was also much broader than the DRIP-seq and p-RPA peaks, which is consistent with the fact that γ-H2AX spreads from DSB sites (Supplementary Fig. 3a). Interestingly, we also noticed that the DRIP-seq, p-RPA, and γ-H2AX signals were enriched in early replicating regions of the human genome determined by Repli-seq^42^ (Supplementary Fig. 3b), which is reminiscent of the early replicating fragile sites described by the Nussenzweig laboratory in mouse cells^43^.

### SRSF1-deficient cells do not phenocopy shTop1 cells defects

**Fig. 4:**
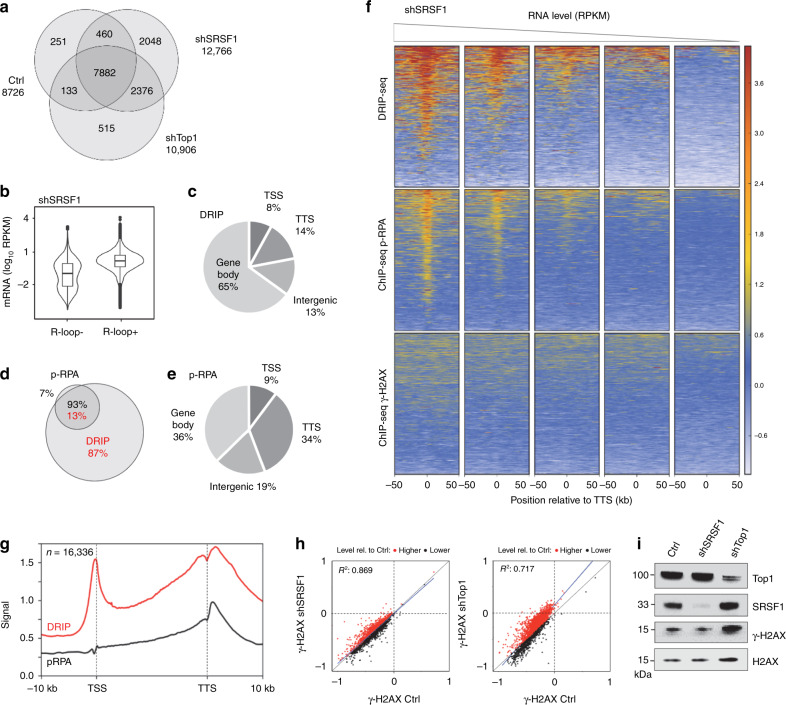
Depletion of SRSF1 increases R-loop and p-RPA at TTS, but not γ-H2AX. **a** Venn diagram of the number of genes enriched in R-loops in control, shSRSF1, and shTop1 cells. R-loop-positive genes correspond to genes overlapping with R-loop peaks identified with MACS2. **b** mRNA level (RPKM) of genes overlapping (R-loop+) or not (R-loop−) with S9.6 peaks in shSRSF1 cells. Box: 25th and 75th percentiles; central line: median. **c** Distribution of R-loop peaks in shSRSF1 cells relative to gene annotations. Peaks were obtained with MACS2 and were analyzed with CEAS (Cis-Regulatory Element Annotation System). **d** Venn diagram of the percentage of genes overlapping with R-loop (red) and p-RPA peaks (black) peaks (MACS2) in shSRSF1 cells. **e** Distribution of p-RPA (S33) peaks in shSRSF1 cells relative to gene annotations. **f** Heat map of the intensity of RNA:DNA hybrids (DRIP), p-RPA, and γ-H2AX at TTS in shSRSF1 cells for five groups of genes with decreasing mRNA levels (RPKM). Within each group, genes were sorted relative to the intensity of DRIP signal. **g** Metaplot of RNA:DNA hybrids (DRIP, red) and p-RPA (black) at 16,336 active genes in shSRSF1 cells. Error bars correspond to SEM. **h** Scatter plot of the intensity of γ-H2AX signal at all active genes in control, shSRSF1, and shTop1 cells. **i** Western blot analysis of γ-H2AX levels on chromatin in control, shSRSF1, and shTop1 cells. H2AX was used as a loading control (*n* = 3).

The accumulation of DSBs and γ-H2AX in shTop1 cells could either be due to R-loops or to topological stress. To discriminate between these possibilities, we depleted the splicing factor SRSF1 in HeLa cells to increase the formation of R-loops without interfering with the relaxation of supercoiled DNA^35,44^. The inducible expression of a shRNA targeting SRSF1 (shSRSF1) in control cells increased R-loop levels and reduced fork speed in an RNase H1-sensitive manner without changing cell-cycle distribution (Supplementary Fig. 4a–d). We also detected an increased level of R-loops at highly expressed genes in shSRSF1 cells, with a distribution similar to those observed in control and shTop1 cells (Fig. 4a–d). Interestingly, shSRSF1 cells also accumulated p-RPA at the TTS of highly expressed genes containing R-loops (Fig. 4d–g), but did not show increased γ-H2AX levels (Fig. 4f, h, i), unlike shTop1 cells (Fig. 3d). Altogether, these data indicate that DSBs form more frequently in shTop1 cells than in shSRSF1 and control cells and suggest that increased R-loops at TTS is necessary but not sufficient for DSB induction.

### DSBs form at TTS containing R-loops in shTop1 cells

**Fig. 5:**
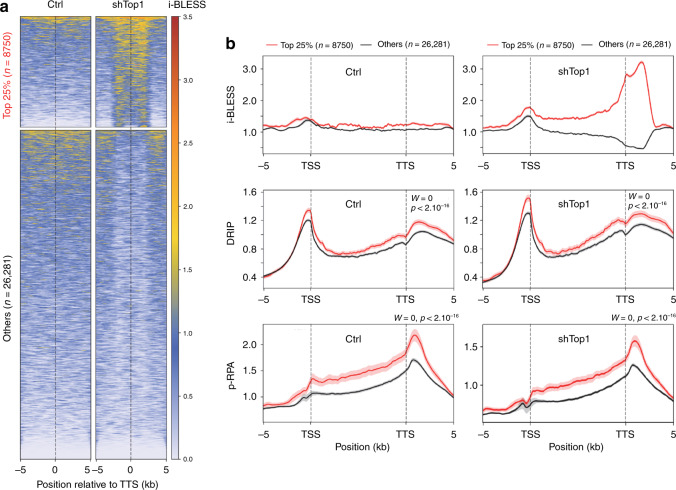
DSBs form at TTS of genes enriched in R-loops and p-RPA in shTop1 cells. **a** Heat map of the intensity of i-BLESS signal at TTS in control and shTop1 cells for two groups of genes determined according to the intensity of i-BLESS signal at the TTS (±2 kb) in shTop1 cells (Top 25%). **b** Metaplots of i-BLESS, RNA:DNA hybrids and p-RPA32 S33 signal for the Top 25% (red) and other (black) genes in control and shTop1 HeLa cells. Error bars indicate SEM. Differences in signal intensity at TTS ±2 kb were calculated with the Wilcoxon rank-sum test with continuity correction.

As the resolution of γ-H2AX ChIP-seq profiles is not sufficient to determine the exact position of chromosome breaks, we next used a next-generation sequencing-based assay called i-BLESS to map DSBs at nucleotide resolution^45,46^. To determine whether shTop1 cells accumulate DSBs at TTS, we measured the intensity of i-BLESS signal for a 2 kb window centered on the TTS of all human genes and sorted them according to the intensity of this signal (Fig. 5a). Interestingly, the TTS of the top 25% genes also showed an increased level of DRIP and p-RPA (Fig. 5b). A similar result was obtained when we used a hierarchical clustering approach to identify genes with increased i-BLESS signal at TTS (DSB+, *n* = 9533) in shTop1 cells (Supplementary Fig. 5a). Again, DSB+ genes showed increased levels of R-loops, p-RPA, and γ-H2AX relative to DSB- genes (Supplementary Figs. 5b and 6a). Interestingly, we also found increased i-BLESS signal at the TSS of a subset of genes in shTop1 cells (Supplementary Fig. 5c), which is consistent with the presence of transcription-dependent DSBs in promoter regions^47^. However, these breaks were not associated with increased p-RPA levels (Supplementary Fig. 5d), unlike DSBs at TTS (Fig. 5b; Supplementary Fig. 5b).

Finally, we analyzed the incidence of gene orientation on DSB formation. Although the percentage of genes in converging (HO) or codirectional (CD) orientations was not significantly different between DSB+ and DSB− genes (44% vs 45% for HO), DSB+ genes showed increased DRIP and p-RPA signals at closely arranged HO genes (<5 kb between TTS) compared to DSB- genes (Supplementary Fig. 6b). This difference was less marked for CD genes (Supplementary Fig. 6c). Altogether, these data indicate that the increased γ-H2AX signal observed in shTop1 cells results from DSBs occurring at the TTS of a large number of genes enriched in R-loops and p-RPA.

## Discussion

It is now well established that R-loops have both positive and negative impacts on genome activity, but the difference between physiological and pathological R-loops has remained unclear. Here, we have compared the distribution of R-loops, replication stress markers (p-RPA and γ-H2AX) and DSBs in HeLa cells to identify R-loops that are detrimental to DNA replication and activate ATR. Using DRIP-seq, we have identified hotspots of R-loop formation at the promoters and terminators of highly expressed genes, as described earlier^22^. Depletion of Top1 further increased R-loop levels at TTS and especially at converging genes, presumably because of the accumulation of topological stress^17^. Interestingly, we found that only 27% of R-loop containing genes colocalized with phospho-RPA32 (S33), a mark of ATR activation used here as a proxy for stalled replication forks. Yet, 84–90% of these p-RPA peaks were associated with R-loops. These values are derived from the conservative analysis of a weak ChIP signal in a population of unchallenged and asynchronously growing cells, so it could be that the actual number of p-RPA peaks is higher. Yet, these data indicate that p-RPA does not accumulate at all R-loops and suggest that only a fraction of R-loop containing genes are responsible for most of the fork pausing events in unchallenged growth conditions. Incidentally, these data indicate that the vast majority of the cotranscriptional R-loops present in the human genome do not interfere with DNA replication or at least do not induce a detectable activation of ATR.

One of the most striking differences between the distribution of DRIP and p-RPA signals is that R-loops were detected at both TSS and TTS of highly expressed genes whereas p-RPA was mostly enriched at TTS. As promoter regions of highly expressed genes usually contain active replication origins^14,15^, this asymmetry in p-RPA distribution may reflect an influence of fork polarity on transcription–replication conflicts^7,8^. A meta-analysis of replication fork direction through 16,336 active genes (RPKM > 0) confirmed that TSS and gene bodies are preferentially replicated codirectionally (RFD+), whereas TTS are mostly replicated by head-on forks (RFD−). Remarkably, p-RPA was enriched at RFD− regions, supporting the view that RPA is phosphorylated by ATR upon fork pausing at TTS enriched in R-loops. Our data are consistent with a recent study showing that R-loops interfere with fork progression in an orientation-dependent manner on a human episomal system^11^ and extend this observation to the genome-wide level. Interestingly, p-RPA enrichment was further increased at the TTS of converging genes, proportionally to the levels of gene expression and to the proximity of the nearest HO-orientation gene neighbor. In addition, p-RPA levels at TTS were increased by the proximity of a replication origin. Altogether, these data suggest that transcription terminators represent hotspots of R-loops and replication fork arrest in the human genome, acting in a context- and orientation-dependent manner.

Top1 depletion in HeLa cells increased levels of γ-H2AX, phospho-RPA32 (S4/S8), and DNA breaks relative to control cells. To determine whether chromosome breaks occur at TTS, we have analyzed the distribution of DSBs at the nucleotide resolution using i-BLESS^46^, an improved version of the original BLESS assay^45^. DSBs were detected downstream of the TTS of a large number of genes that were also enriched in R-loops and p-RPA, especially in regions of the genome where transcription converges. Since it has been recently reported that replication forks blocked by R-loops can be restarted by fork cleavage in a MUS81-dependent manner^48^, an attractive possibility could be that DSBs detected at TTS are generated by structure-specific endonucleases. Interestingly, DSBs were also detected upstream of TSS, which may correspond to the replication-independent DSBs identified at promoter regions in other studies^47^. Recent reports indicate that these DSBs may depend on Topoisomerase IIβ activity and on the proximity of CTCF sites at loop anchors^49,50^. These breaks could be distinct from the replication-dependent DSBs occurring at TTS, which could be more related to the estrogen-induced DSBs occurring during S phase at R-loop-containing genes in breast cancer cells^51^.

**Fig. 6:**
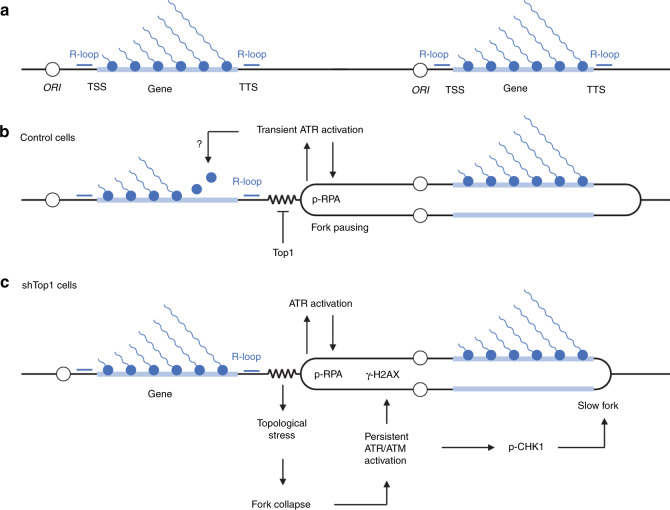
Model of the regulation of TRCs in the human genome. **a** Highly expressed genes form co-transcriptional R-loops at TSS, TTS and to a lesser extent in gene bodies. Replication origins are frequently located upstream of TSS. **b** Initiation from upstream origins ensures that R-loops at TSS and gene bodies are preferentially replicated codirectionally, which would limit HO conflicts. Forks progressing in the opposite direction pause when they encounter the TTS of highly expressed genes, presumably because of the accumulation of positive supercoiling. Transient fork pausing activates ATR and leads to the phosphorylation of RPA32 on S33. ATR may also promote the displacement of RNA polymerases ahead of the paused fork. **c** In the absence of Top1, the accumulation of torsional stress may lead to fork collapse and to the sustained activation of ATR/ATM. This would in turn slowdown fork progression throughout the genome.

An important question that remains to be addressed is the mechanism by which R-loops interfere with DNA replication in human cells. It is generally proposed that RNA:DNA hybrids are intrinsically difficult to replicate and impede fork progression in an orientation-dependent manner. However, our DNA fiber analyses revealed that all replication forks were equally slowed down by 30–40% in shTop1 cells, which argues against a direct effect of R-loops. Indeed, highly expressed genes cover only a small fraction of the human genome and R-loops should therefore affect only a subset of forks in shTop1 cells. This should lead to a bimodal distribution of CldU track lengths and not a global reduction of fork speed. We rather favor a model in which replication fork pausing at TTS prevents HO collisions with transcription (Fig. 6). Top1-deficient cells could experience difficulties to stabilize these paused forks, which would increase the risk of fork collapse and DSB formation, presumably in a MUS81-dependent manner^52^. DSBs would in turn induce a chronic activation of S-phase checkpoints and a slowdown of replication forks. This view is supported by the fact that cells depleted for the splicing factor SRSF1, which have increased R-loop levels but no DNA relaxation problems, have faster replication forks and less γ-H2AX than shTop1 cells. This model is consistent with reports showing that ATR downregulates elongation at undamaged forks in yeast and human cells^53–55^. It is also consistent with data in budding yeast showing that fork arrest does not directly depend on R-loops and is mechanistically separable from the induction of DNA damage^56^. Yet, it is worth noting that the overexpression of RNase H1 partially rescued the slow fork phenotype of shTop1 cells, suggesting that RNA:DNA hybrids still have a negative impact on DNA replication in these cells. To explain this apparent discrepancy, we propose that RNA:DNA hybrids form at stalled forks as a consequence of fork arrest and could impede fork restart. This would be reminiscent of the formation of RNA:DNA hybrids at DSBs, which interfere with HR-mediated repair^57^.

In conclusion, our results suggest that polar fork arrest at TTS is an active process that prevents collisions between RNA and DNA polymerases, as previously reported in budding yeast^58,59^. Transient fork pausing could help cells displace RNA polymerases ahead of the replisome, through a process involving Mec1 and INO80^60,61^. As transcription is a discontinuous process^62,63^, forks may also pause during transcription bursts and restart after passage of RNA polymerase convoys. In this model, TTS could act as traffic lights, arresting forks until road blocks have been removed. Alterations of DNA relaxation or pre-mRNA cleavage could perturb this coordination, leading to increased DNA breaks and to the chronic activation of ATR^35,64^, which would reduce in turn the speed of replication forks. Our data are consistent with recent models in which initiation of DNA replication upstream of highly expressed genes would facilitate the coordination between replication and transcription^14,15^. This is reminiscent of the codirectional organization of genes in *B. subtilis* and other bacteria to avoid head-on conflicts with replication^8,12^. This organization does not exist in budding yeast, in which persistent R-loops were recently shown to cause genomic instability independently of their orientation^65^. In metazoan, this organization would accommodate extensive changes in gene expression profiles occurring during cell differentiation. The functional coupling between strong origins and promoters would therefore represent a simple and flexible mean to limit transcription–replication conflicts in differentiating cells. Interestingly, it has been recently reported that the deregulation of oncogenic pathways activates intragenic replication origins that induce HO conflicts and chromosome breaks^66^. It is therefore tempting to speculate that the loss of a functional organization restraining replication–transcription conflicts to TTS leads to genomic instability in precancerous lesions.

## Methods

### Cell culture

HeLa, HEK293T and *Asi*SI–ER-U2OS cells were cultured in Dulbecco’s modified Eagle’s medium (DMEM) supplemented with 10% fetal calf serum (FCS) and 100 U ml^−1^ penicillin/streptomycin at 37 °C in 5% CO_2_.

### Production of lentiviral vectors and cell transduction

HIV-1-derived lentiviral vectors were produced in HEK293T cells^67^. To this end, cells were seeded on poly-D-lysine coated plates and transfected with packaging plasmid (psPAX2, Addgene plasmid #12260): transfer vector (pLVX-Tet-on; TRIPZ-shTop1): vesicular stomatitis virus envelop plasmid (pMD2.G, plasmid #12259) at a ratio 5:3:2 by the calcium phosphate method. The culture medium was collected 48 h post-transfection, filtrated using 0.45-μm filters and concentrated at 100 folds by ultracentrifugation at 89,000 × *g* at 4 °C for 1.5 h. HeLa cells were transduced at a MOI = 10 (multiplicity of infection) by centrifugation at 1500 × *g* at 30 °C for 90 min in the presence of 5 μg ml^−1^ of Polybrene.

### DNA fiber spreading

To perform DNA fiber spreading^68^, HeLa control and shTop1 HeLa cells were treated with 2 µg ml^−1^ doxycycline for 24 h and then transfected with the plasmid EGFP-N1 or RNase H1-EGFP (see Supplementary Table 1) for 48 h in the presence of doxycycline. Subconfluent cells were sequentially labeled first with 10 µM 5-iodo-2′-deoxyuridine (IdU) and then with 100 µM 5-chloro-2′-deoxyuridine (CldU) for the indicated times. One thousand cells were loaded onto a glass slide (StarFrost) and lysed with spreading buffer (200 mM Tris-HCl pH 7.5, 50 mM EDTA, 0.5% SDS) by gently stirring with a pipette tip. The slides were tilted slightly and the surface tension of the drops was disrupted with a pipette tip. The drops were allowed to run down the slides slowly, then air dried, fixed in methanol/acetic acid 3:1 for 10 min, and allowed to dry. Glass slides were processed for immunostaining with mouse anti-BrdU to detect IdU, rat anti-BrdU to detect CldU, mouse anti-ssDNA antibodies (see Supplementary Table 1 for details), and corresponding secondary antibodies conjugated to various Alexa Fluor dyes. Nascent DNA fibers were visualized using immunofluorescence microscopy (Leica DM6000 or Zeiss ApoTome). The acquired DNA fiber images were analyzed by using MetaMorph Microscopy Automation and Image Analysis Software (Molecular Devices) and statistical analysis was performed with GraphPad Prism (GraphPad Software). The length of at least 150 CldU tracks were measured per sample.

### Detection of pRPA32-S4/S8 foci by immunofluorescence

Cells growing on coverslips were incubated for 3 min at room temperature with CSK buffer (10 mM PIPES, pH 7.0; 100 mM NaCl; 3 mM MgCl_2_; 300 mM sucrose and 0.3 mg ml^−1^ RNase A) containing 0.7 % Triton X-100 and phosphatase inhibitor cocktail and fixed with 2 % PFA for 10 min at room temperature. The coverslips were incubated with an anti-pRPA32-S4/S8 antibody overnight at 4 °C and then with a secondary antibody conjugated to an Alexa Fluor dye for 1 h at 37 °C, followed by DAPI staining and ProlongGold mounting. Images were acquired by using a Zeiss LSM780 confocal or a Zeiss ApoTome microscope. The mean fluorescence intensity (MFI) in cells was quantified by using CellProfiler (www.cellprofiler.org).

### Detection of RNA:DNA hybrids by slot blotting

Cells were lysed in 0.5% SDS/TE, pH 8.0 containing Proteinase K overnight at 37 °C. Total DNA was isolated with phenol/chloroform/isoamylalcohol extraction followed by standard ethanol precipitation and quantified using Nanodrop. Half microgram of total DNA was loaded in duplicate onto a Hybond-N^+^ membrane using slot blot apparatus. The membrane was separated in two, one for direct UV crosslinking at 0.12 Joules and the other for DNA denaturation. To denature DNA, membrane was incubated with denaturation buffer (0.5 M NaOH; 1.5 M NaCl) for 10 min and neutralization buffer (1 M NaCl and 0.5 M Tris, pH 7.5) for another 10 min prior to UV crosslinking. Membranes were blocked with 5% skim milk in PBST (PBS; 0.1% Tween-20) for 1 h. The RNA:DNA hybrids and ssDNA were detected by immunoblotting.

### Chromatin fractionation

Cells were incubated with CSK-Triton lysis buffer (10 mM PIPES, pH6.8; 100 mM NaCl; 1 mM MgCl_2_; 1 mM EGTA; 300 mM Sucrose; 10 mM DTT; 0.2% Triton X-100; protease inhibitor; phosphatase inhibitor) on ice for 10 min and harvested by scraping. The supernatant was collected after centrifugation at 0.8 × *g* for 5 min at 4 °C. Pellet was resuspended in CSK-Triton buffer and incubated for 10 min on ice. Another round of centrifugation at 0.8 × *g* for 5 min at 4 °C was performed to separate nucleoplasm and chromatin fractions, supernatant and pellet, respectively. Uncropped original scans of western blots are shown in Supplementary Fig. 7.

### Chromatin immunoprecipitation sequencing (ChIP-seq)

Formaldehyde was added to the culture medium to a final concentration of 1% for 10 min at room temperature. Glycine was added to a final concentration of 0.125 M for 5 min to stop crosslinking. Cells were harvested by scraping after PBS wash. Pelleted cells were lysed in lysis buffer (50 mM PIPES, pH 8; 85 mM KCl; 0.5% NP-40). The lysates were homogenized with a Dounce homogenizer and nuclei were harvested by centrifugation. Nuclei were then incubated in nuclear lysis buffer (50 mM Tris, pH 8.1; 10 mM EDTA; 1% SDS) and sonicated at 70% amplitude for a duration of 3 min and 25 s with 15 s on and 45 s off (Qsonica Q700 sonicator) to obtain DNA fragments of about 500–1000 bp. Samples were diluted 10 times in dilution buffer (0.01 % SDS; 1.1% Triton X-100; 1.2 mM EDTA; 16.7 mM Tris, pH 8.1; 167 mM NaCl) and subjected to a 45 min preclearing with 140 μl of previously blocked protein-A and protein-G beads. Blocking was achieved by incubating the agarose beads with 500 μg of BSA and 200 μg of herring sperm DNA for 3 h at 4 °C. Precleared samples were incubated overnight at 4 °C with antibodies specific for γ-H2AX (10 μl) or without antibody as negative control. Immune complexes were then recovered by incubating the samples with 140 μl of blocked protein-A/protein-G beads for 2 h at 4 °C on a rotating wheel. Beads were washed once in dialysis buffer (2 mM EDTA; 50 mM Tris, pH 8; 0.2% Sarkosyl) and four times in wash buffer (100 mM Tris, pH 8.8; 500 mM LiCl; 1% NP-40; 1% NaDoc). Elution from the beads was achieved by incubation in elution buffer (1% SDS; 100 mM NaHCO_3_) for 15 min. Crosslink was reversed by adding 0.2% SDS and RNase A to the samples and incubating overnight at 70 °C. After a 2-h proteinase K treatment, DNA was precipitated by phenol/chloroform extraction and ethanol precipitation. The AsiSI–ER-U2OS cells treated with or without 4-hydroxytamoxifen (4-OHT) were included as positive control for the validation of experiments^69^. The pulled down material and input DNA were then size-selected, and ligated to Illumina barcoded adaptors, using TruSeq ChIP Sample Preparation Kit (Illumina) or ThruPLEX® DNA-seq Kit (Rubicon Genomics) for next-generation sequencing (NGS) on Illumina HiSeq 2500 and HiSeq 4000 platforms.

For phospho-RPA2-S33 ChIP, similar procedure was performed with minor modifications. Cells were resuspended in sonication buffer (50 mM HEPES, pH 8.0; 140 mM NaCl; 1 mM EDTA; 1% Triton X-100; 0.1% NaDoc; 0.5% SDS) and proceeded to sonication. Immunoprecipitation was performed using 30 μg chromatin and 4 μg anti-phospho-RPA2-S33 antibody. The pulldown material was eluted using IPure kit (Diagenode) and proceeded to NGS as described above.

### DNA–RNA immunoprecipitation sequencing (DRIP-seq)

To perform DRIP-seq^70^, cells (5 × 10^6^) were lysed in 0.5% SDS/TE, pH 8.0 containing Proteinase K overnight at 37 °C. Total DNA was isolated with phenol/chloroform/isoamylalcohol extraction followed by standard ethanol precipitation. One-third of total DNA was fragmented by a cocktail of restriction enzymes (*Eco*RI, *Hin*dIII, *Bsr*gI, *Ssp*I, *Xba*I) overnight at 37 °C. A negative control treated overnight with RNase H was included. Digested DNA was purified by phenol/chloroform/isoamylalcohol extraction, ethanol precipitation and quantified by Nanodrop. Four micrograms of digested DNA were diluted in binding buffer (10 mM NaPO_4_, pH 7.0; 0.14 M NaCl; 0.05% Triton X-100) and incubated with 10 μg of S9.6 antibody overnight at 4 °C on a rotator. DNA/antibody complexes were added for 2 h at 4 °C to Agarose Protein-A/G beads prewashed with binding buffer. Immunoprecipitated DNA was eluted by incubating with elution buffer (50 mM Tris pH 8.0; 10 mM EDTA; 0.5% SDS) containing Proteinase K at 55 °C for 45 min on a rotator. The eluent was precipitated by phenol/chloroform/isoamylalcohol extraction and ethanol precipitation. Validation of DRIP procedure was performed by qPCR (see Supplementary Table 2 for primer sequences). The pulled down material and input DNA were then sonicated, size-selected, and ligated to Illumina barcoded adaptors, using TruSeq ChIP Sample Preparation Kit (Illumina) or ThruPLEX® DNA-seq Kit (Rubicon Genomics) for next-generation sequencing (NGS) on Illumina HiSeq 2500 platform.

### RNA-seq

RNA sequencing (RNA-seq) libraries were prepared using the Illumina TruSeq Stranded mRNA Library Prep Kit. Paired-end RNA-seq were performed with an Illumina NextSeq sequencing instrument (Helixio, France).

### i-BLESS

Samples for i-BLESS analysis were prepared as described^46^ with minor modifications. Approximately 10 million of HeLa cells were resuspended in PBS buffer and mixed with 1% low melting point agarose in PBS buffer at 40 °C. Cell suspension was mixed with liquid paraffin at 40 °C and vigorously shaken by hand for 1 min, until emulsion was formed. The emulsion was then poured into ice-cold PBS buffer and the mixture was stirred for several minutes. Agarose bead suspension was gently centrifuged (200 × *g*, 10 min), paraffin layer was removed and agarose bead pellet was washed 3 times with TE buffer. Beads were washed with ES buffer (1% Sarkosyl, 25 mM EDTA, pH 8.0), resuspended in ES with 50 µg ml^−1^ Proteinase K and incubated overnight at 50 °C. After incubation, the beads were washed with TE + 0.1 mM PMSF and twice with TE. Next, the beads were washed in 1 × Blunting Buffer (NEB), followed by DNA ends blunting using Quick Blunting kit (NEB) for 2 h and then washed twice with TE. The beads were subsequently washed with dA-Tailing Reaction Buffer (NEB) and DNA ends were A-tailed using NEBNext® dA-Tailing Module for 80 min. Next, the beads were washed with T4 ligation buffer and then resuspended in T4 ligation buffer with 100 nM P5 adapter and T4 ligase (NEB) and incubated overnight at 16 °C. After ligation, the beads were washed once with TE, and encapsulated DNA was initially sonicated using Covaris S220. Total DNA was isolated using Zymoclean™ Large Fragment DNA Recovery Kit (Zymo Research) and once again fragmented by sonication to create ~400 bp fragments. Labeled fragments were captured by streptavidin beads (Invitrogen), blunted and A-tailed using NEBNext® Ultra™ End Repair/dA-Tailing, then ligated to a P7 adapter. The resulting circular DNA was then linearized by USER (NEB) digestion and amplified and indexed by PCR using Illumina PCR primers. Quality and quantity of the resulting libraries were assessed on 2100 Bioanalyzer using HS DNA Kit (Agilent), and on Qubit 2.0 Fluorometer using Qubit dsDNA HS Assay Kit (Life Technologies). The libraries were sequenced (1 × 61 bp) on Illumina HiSeq 2500 platform, according to the modified experimental and software protocols for generation of high-quality data for low-diversity samples^71^.

### Comet assay

DNA breaks were monitored using the OxiSelect Comet Assay Kit (CELL BIOLABS, Inc.) according to the manufacturer’s instructions. Slides were visualized using immunofluorescence microscopy (Zeiss ApoTome). The acquired comet images were analyzed by using MetaMorph Microscopy Automation and Image Analysis Software (Molecular Devices) and statistical analysis was performed with GraphPad Prism (GraphPad Software). A total of 200 cells were analyzed.

### Bioinformatic analyses

The quality of sequencing data was assessed with FastQC (http://www.bioinformatics.babraham.ac.uk/projects/fastqc) and in-house PERL and Python scripts. ChIP-seq and DRIP-seq data were aligned to Human genome reference (hg19 assembly) with Bowtie2^72^ and RNA-seq using STAR^73^. Mapping quality was assessed with SAMtools^74^ and in-house Python scripts. Peak-calling for DRIP-seq data was done using MACS2^75^ with a *q*-value of 0.05 and keeping up to five replicates. Reproducible peaks from replicates were then selected using the Irreproducible Discovery Rate (IDR) method from ENCODE Project^76^, with a cutoff value of 0.05. Only the expressed genes, with the transcription RPKM > 0, were selected to determine the impact of different gene positions on R-loop formation. Intersection of transcripts annotation (RefSeq, hg19) with R-loop signal was done using BEDTools^77^. The analyses of replication fork directionality and replication initiation zones used the published OK-seq data from HeLa cells^14^. DeepTools2^78^ was used to compute and draw enrichment heat maps and profiles on positions of interest (peaks, TSS, TTS). Further analyses were done in R (http://www.R-project.org), with Bioconductor packages and ggplot2 for graphic representation^79^.

### Reporting summary

Further information on research design is available in the Nature Research Reporting Summary linked to this article.

## Supplementary information

Supplementary information

Peer review

Reporting summary

## Data Availability

The data sets generated and/or analyzed during the current study are available from the corresponding authors on reasonable request. The NGS data sets generated and analyzed during the current study are available in the GEO repository, accession number: GSE108172.
