# Supplementary material for: Topoisomerase 1 prevents replication stress at R-loop-enriched transcription termination sites
Source: Nat Commun. 2020 Aug 7;11:3940. doi: 10.1038/s41467-020-17858-2 (PMC7414224; doi:10.1038/s41467-020-17858-2)
Supplement: Supplementary file 1 — Supplementary information [file 41467_2020_17858_MOESM1_ESM.pdf]

Topoisomerase 1 prevents replication stress at R-loop-enriched transcription termination sites

Alexy Promonet*, Ismaël Padioleau*, Yaqun Liu*, Lionel Sanz, Anna Biernacka, Anne-Lyne Schmitz, Magdalena Skrzypczak, Amélie Sarrazin, Clément Mettling, Maga Rowicka, Krzysztof Ginalski, Frédéric Chedin, Chun-Long Chen, Yea-Lih Lin and Philippe Pasero

Supplementary Information

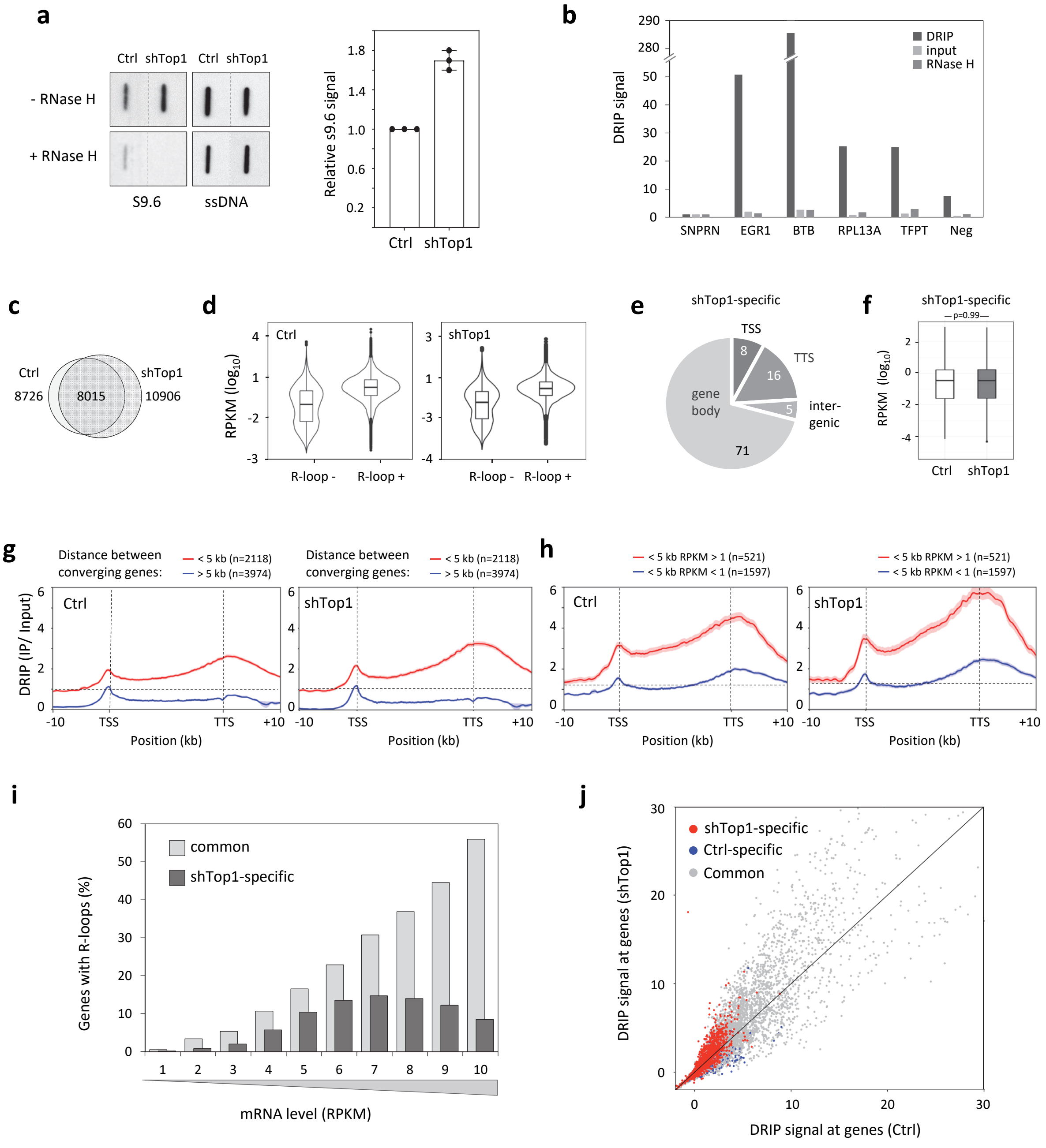

Supplementary Figure 1. Depletion of Top1 in HeLa cells increases the formation of R-loops

(**a**) Relative amount of RNA:DNA hybrids in control and shTop1 cells as determined by slot blot analysis with the S9.6 antibody before and after RNase H treatment. Error bars correspond to SD of three independent experiments. (**b**) DRIP-qPCR analysis of the relative enrichment of RNA:DNA hybrids at five genes used here as positive (TFPT, RPL13, BTB, EGR1) and negative (SNRPN, Neg) controls, before and after RNase H treatment. (**c**) Venn diagram of the number of genes enriched in R-loops in control and shTop1 cells. R-loop positive genes correspond to genes overlapping with R-loop peaks identified with MACS2. (**d**) mRNA level (RPKM) of genes overlapping (R-loop positive) or not (R-loop negative) with S9.6 peaks in control and shTop1 cells. Box: 25th and 75th percentiles; central line: median. (**e**) Genomic distribution of R-loop peaks (MACS2) at genes that are specific to shTop1 cells. (**f**) mRNA level (RPKM) for genes containing R-loops in shTop1 cells, but not in control cells. Box: 25th and 75th percentiles; central line: median. P-values were calculated with Student's t-test. (**g**) Metaplots of the distribution of S9.6 signals at converging genes depending on the distance between their TTS in control and shTop1 HeLa cells. Data are presented as mean values +/- SEM. (**h**) Metaplots of the distribution of S9.6 signal at converging genes spaced by less than 5 kb relative to mRNA levels in control and shTop1 HeLa cells. Data are presented as mean values +/- SEM. (**i**) Human genes were sorted according to mRNA level and were distributed in deciles of increasing RPKM. The proportion of genes only containing R-loops in shTop1 cells (dark grey) or in all cell types (light grey) is shown. (**j**) Scatter plot of DRIP signal intensity at genes in control and shTop1 cells. Genes that only contain R-loops in shTop1 cells are colored in red and genes that only contain R-loops in control cells are marked in blue. Grey dots correspond to genes that contain R-loops in both cell types.

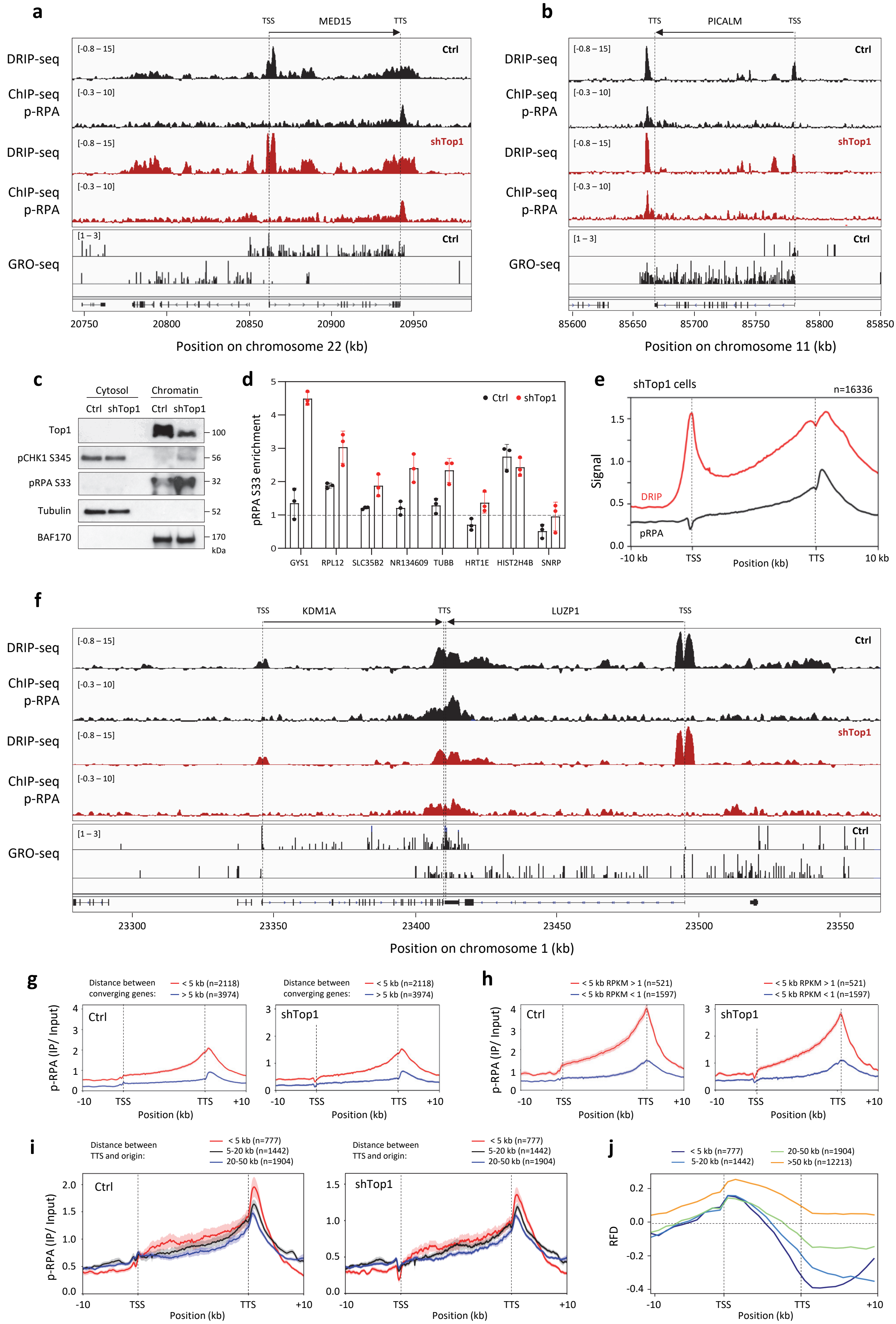
a
TSS
MED15
TTS
DRIP-seq
ChIP-seq p-RPA
DRIP-seq
ChIP-seq p-RPA
GRO-seq
[-0.8 – 15]
[-0.3 – 10]
[1 – 3]
Ctrl
shTop1
20750 20800 20850 20900 20950
Position on chromosome 22 (kb)
b
TTS
PICALM
TSS
85600 85650 85700 85750 85800 85850
Position on chromosome 11 (kb)
c
Cytosol
Chromatin
Ctrl shTop1 Ctrl shTop1
Top1
pCHK1 S345
pRPA S33
Tubulin
BAF170
100
56
32
52
170
kDa
d
pRPA S33 enrichment
Ctrl shTop1
GYS1 RPL12 SLC35B2 NR134609 TUBB HRT1E HIST2H4B SNRP
e
shTop1 cells
n=16336
Signal
DRIP
pRPA
-10 kb TSS Position (kb) TTS 10 kb
f
TSS
KDM1A
TTS
LUZP1
TSS
23300 23350 23400 23450 23500 23550
Position on chromosome 1 (kb)
g
Distance between converging genes:
< 5 kb (n=2118)
> 5 kb (n=3974)
p-RPA (IP/ Input)
Ctrl
shTop1
-10 TSS TTS +10
Position (kb)
h
< 5 kb RPKM > 1 (n=521)
< 5 kb RPKM < 1 (n=1597)
i
Distance between TTS and origin:
< 5 kb (n=777)
5-20 kb (n=1442)
20-50 kb (n=1904)
j
< 5 kb (n=777)
5-20 kb (n=1442)
20-50 kb (n=1904)
>50 kb (n=12213)
RFD
-10 TSS TTS +10
Position (kb)

Supplementary Figure 2. The accumulation of p-RPA at TTS depends on the orientation and the level of gene expression

(**a**) Distribution of RNA:DNA hybrids (DRIP-seq) and p-RPA32 S33 (ChIP-seq) at the MED15 gene on chromosome 22 in control and shTop1 HeLa cells. GRO-seq data are shown for HeLa cells. (**b**) Distribution of RNA:DNA hybrids (DRIP-seq) and p-RPA32 S33 (ChIP-seq) at the PICALM gene on chromosome 11 in control and shTop1 HeLa cells. GRO-seq data are shown for HeLa cells. (**c**) Western blot analysis of p-CHK1 (S345) and p-RPA (S33) in the chromatin and cytosolic fractions of control and shTop1 cells. Tubulin and BAF170 are used as loading controls for the cytosolic and chromatin fractions, respectively (n=3). (**d**) ChIP-qPCR analysis of p-RPA (S33) enrichment at the TTS of 7 representative genes in control and shTop1 cells. Error bars correspond to SD of three independent experiments. SNRP is used as a negative control. (**e**) Metaplots of RNA:DNA hybrids and p-RPA for 16336 active genes in shTop1 cells. Error bars correspond to SEM. (**f**) Distribution of RNA:DNA hybrids (DRIP-seq) and p-RPA32 S33 (ChIP-seq) at the converging genes KDM1A and LUZP1 on chromosome 1 in control and shTop1 HeLa cells. GRO-seq data are shown for HeLa cells. (**g**) Metaplots of p-RPA signals at converging genes in control and shTop1 cells depending on the distance between their TTS. Error bars correspond to SEM. (**h**) Metaplots of p-RPA signals at closely spaced converging genes (< 5 kb) in control and shTop1 cells depending on their level of expression (RPKM > or < 1). Error bars correspond to SEM. (**i**) Metaplots of p-RPA signals at genes in control and shTop1 cells depending on the distance between TTS and the next downstream replication origins. Error bars correspond to SEM. (**j**) Impact on replication fork direction (RFD) of the distance between genes and downstream origins.

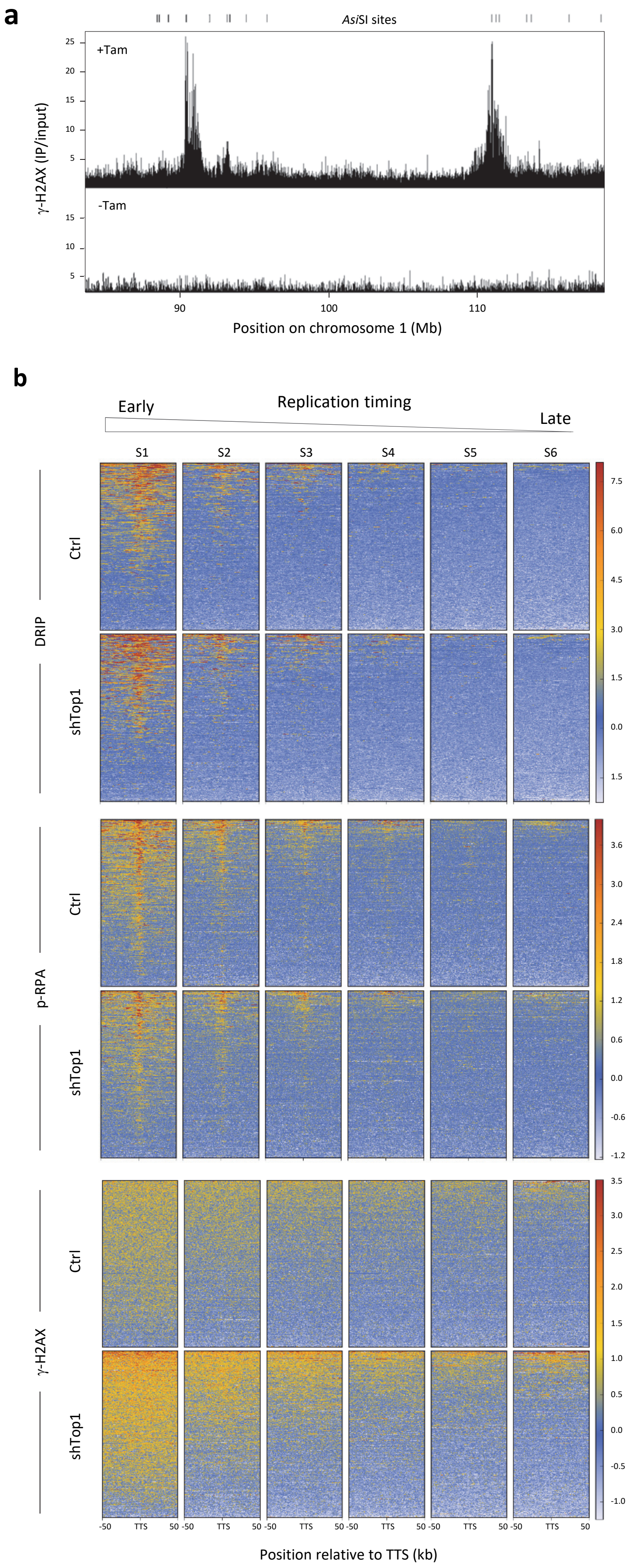
a
AsiSI sites
+Tam
-Tam
γ-H2AX (IP/input)
25
20
15
10
5
90
100
110
Position on chromosome 1 (Mb)
b
Replication timing
Early
Late
S1
S2
S3
S4
S5
S6
DRIP
Ctrl
shTop1
p-RPA
Ctrl
shTop1
γ-H2AX
Ctrl
shTop1
7.5
6.0
4.5
3.0
1.5
0.0
1.5
3.6
3.0
2.4
1.8
1.2
0.6
0.0
-0.6
-1.2
3.5
3.0
2.5
2.0
1.5
1.0
0.5
0.0
-0.5
-1.0
-50
TTS
50
Position relative to TTS (kb)

Supplementary Figure 3. γ-H2AX spreads over megabase domains and accumulates at early replicating regions in shTop1 cells.

(**a**) ChIP-seq analysis of γ-H2AX in U2OS after induction of *Asi*SI breaks (+Tam) using the DIvA system[41], used here as a positive control. A representative region on chromosome 1 is shown. (**b**) Impact of replication timing on the intensity of DRIP, p-RPA and γ-H2AX signals at TTS in control and shTop1 HeLa cells. Replication timing from S1 (early S) to S6 (late S) is derived from published Repli-seq datasets in HeLa cells[42].

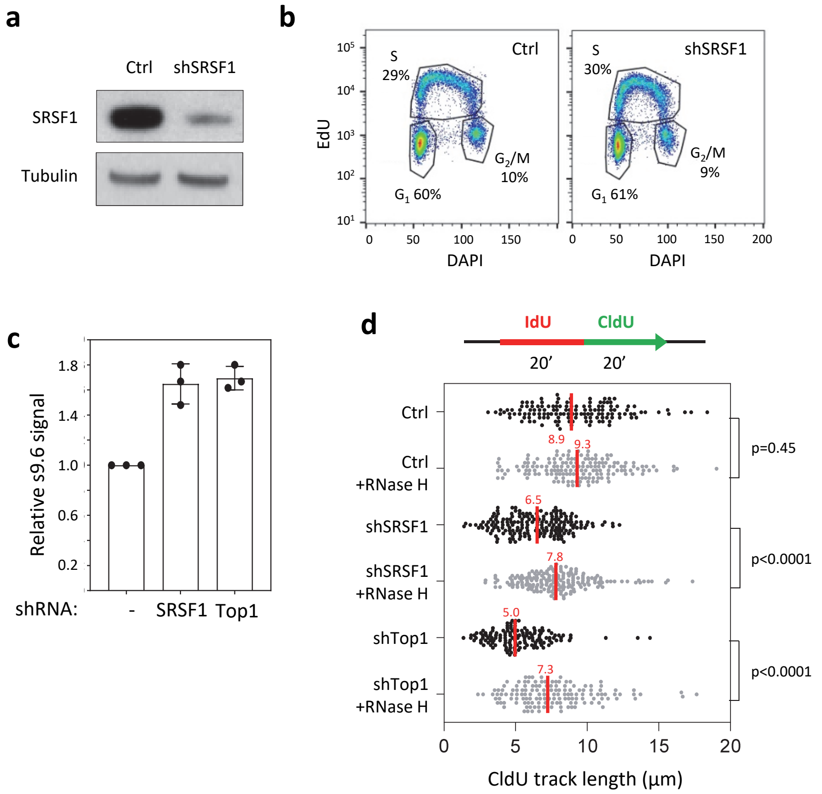

Supplementary Figure 4. Cells depleted for SRSF1 show increased R-loops and p-RPA at TTS, but no increased γ-H2AX

(**a**) Western blot analysis of SRSF1 levels in control HeLa cells and in cells expressing shRNAs targeting SRSF1 (shSRSF1) under the control of a doxycycline-inducible promoter at 72 hours post-induction (n=5). (**b**) The cell cycle distribution of shSRSF1 cells was determined by flow cytometry after labeling of S-phase cells with EdU. The fraction of cells in the different cell cycle phases is indicated. (**c**) Relative amount of RNA:DNA hybrids in control, shSRSF1 and shTop1 cells as determined by slot blot analysis with the S9.6 antibody after RNase H treatment. Error bars correspond to SD of three independent experiments. (**d**) Doxycycline-treated control, shSRSF1 and shTop1 cells were transfected for 48 hours with a mock vector (EGFP-N1) or human RNase H1-EGFP (+RNase H1) and were sequentially labeled with IdU and CldU for 20 minutes. Replication fork progression was measured using DNA fiber spreading as described in Methods. The median length of CldU tracks is indicated in red. At least 150 fibers of each sample were measured (n=3). P-values were calculated with the two-sided Mann-Whitney rank sum test.

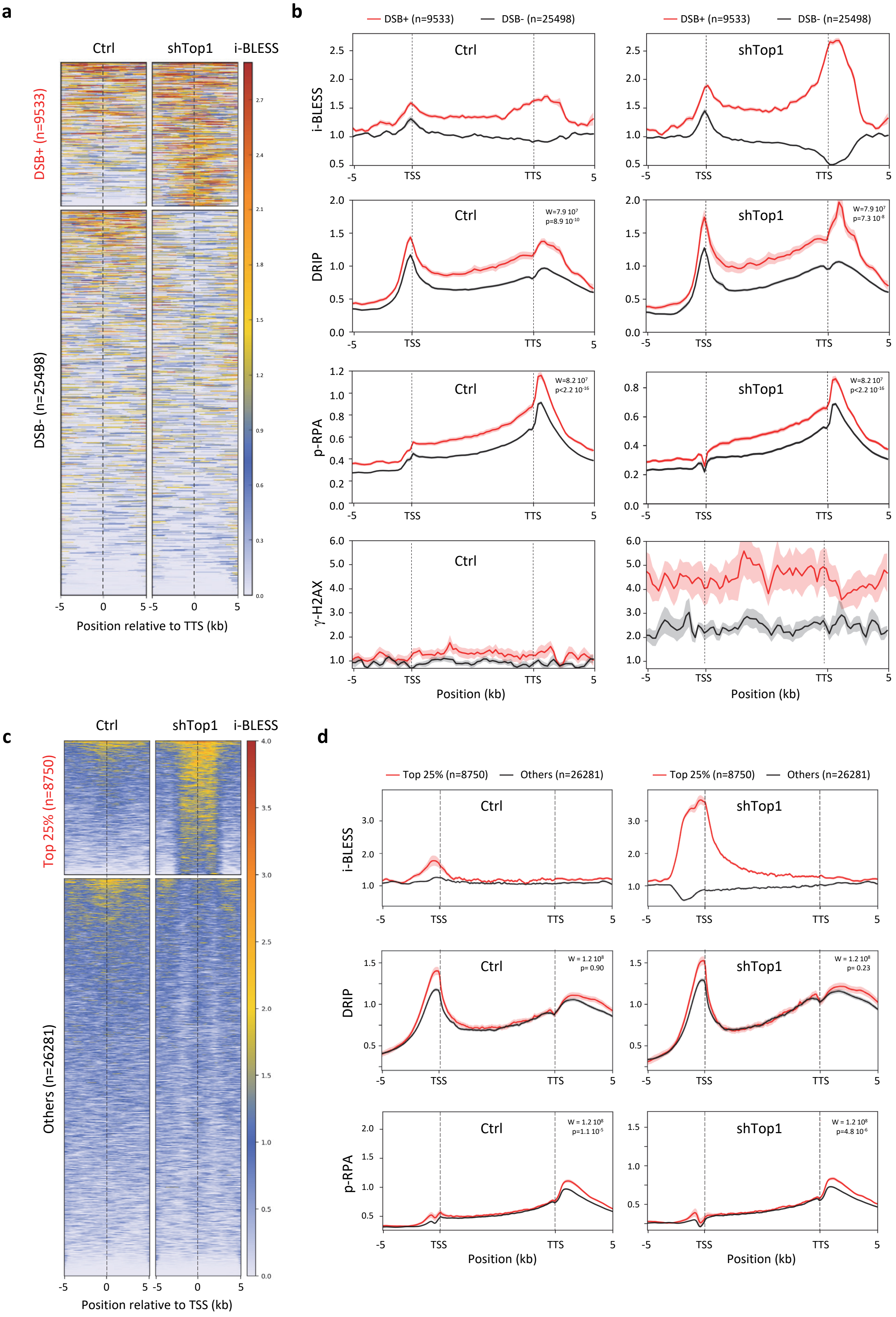
a
b
c
d
Ctrl
shTop1
i-BLESS
DSB+ (n=9533)
DSB- (n=25498)
Position relative to TTS (kb)
DRIP
p-RPA
γ-H2AX
TSS
TTS
Position (kb)
W=7.9 10^7
p=8.9 10^-10
p=7.3 10^-8
W=8.2 10^7
p<2.2 10^-16
Top 25% (n=8750)
Others (n=26281)
Position relative to TSS (kb)
W = 1.2 10^8
p= 0.90
p= 0.23
p=1.1 10^-5
p=4.8 10^-6

Supplementary Figure 5. Top1 prevents DNA breaks at TTS

(**a**) Heat map of the intensity of i-BLESS signal at TTS in control and shTop1 cells for two groups of genes (DSB+ and DSB-) determined by hierarchical clustering analysis of i-BLESS signal at the TTS in shTop1 cells. (**b**) Metaplots of i-BLESS, RNA:DNA hybrids, p-RPA32 S33 and γ-H2AX signal for DSB+ (red) and DSB- (black) genes in control and shTop1 cells, shadows indicate standard error. Differences in signal intensity at TTS +/- 2 kb were calculated with the Wilcoxon rank-sum test with continuity correction. (**c**) Heat map of the intensity of i-BLESS signal at TSS in control and shTop1 cells for two groups of genes determined according to the intensity of i-BLESS signal at the TSS (+/- 2 kb) in shTop1 cells. (**d**) Metaplots of i-BLESS, RNA:DNA hybrids and p-RPA32 S33 signal for the Top 25% (red) and others (black) genes in control and shTop1 HeLa cells. Data are presented as mean values +/- SEM. Differences in signal intensity at TTS +/- 2 kb were calculated with the Wilcoxon rank-sum test with continuity correction.

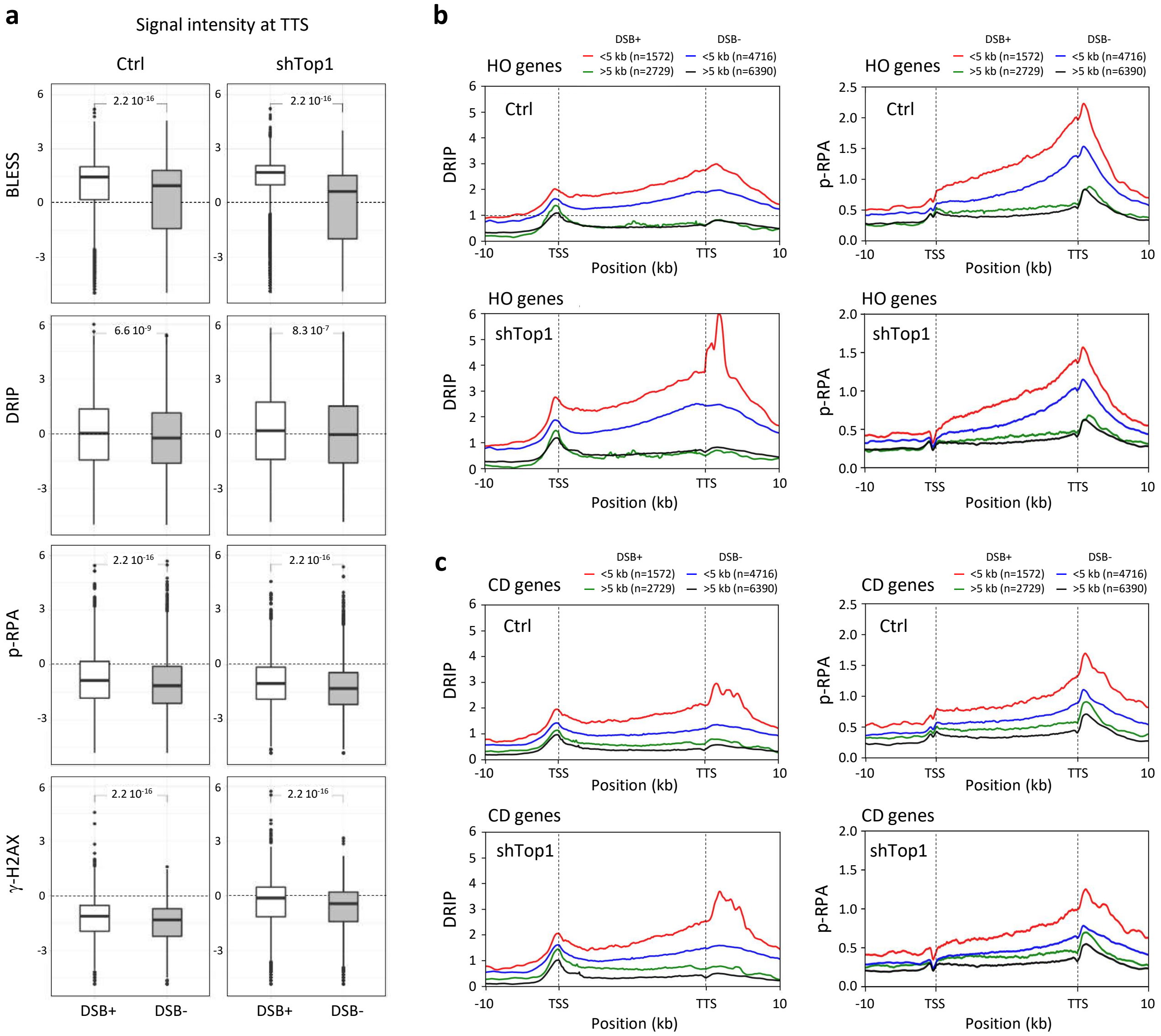

Supplementary Figure 6. i-BLESS, DRIP, p-RPA and γ-H2AX signal intensities at TTS of HO and CD DSB+ and DSB- genes

(**a**) Distribution of i-BLESS, DRIP, p-RPA, γ-H2AX signal intensities at all TTS of DSB+ (white) and DSB- genes (grey) as determined by the clustering analysis of i-BLESS signal in control and shTop1 cells shown in Supplementary Fig. 5a. Box: 25th and 75th percentiles; central line: median. P-values were calculated with a two-sided Student's t-test. (**b, c**) Intensity of DRIP and p-RPA signals at converging (HO) and codirectional (CD) genes from DSB+ and DSB- genes (Supplementary Fig. 5a) and separated by more or less than 5 kb in control and shTop1 cells.

.

Supplementary Figure 7. Uncropped Original Scans

Fig. 1a

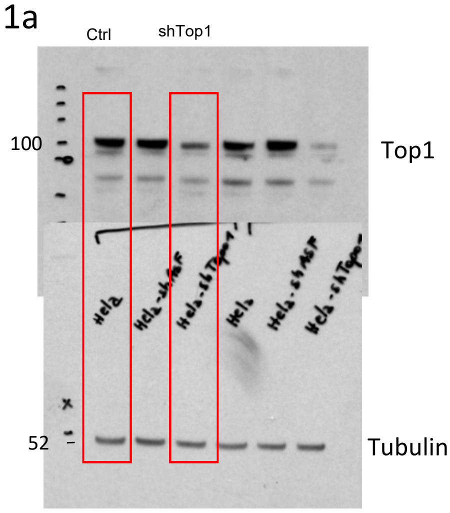

Fig. 3a

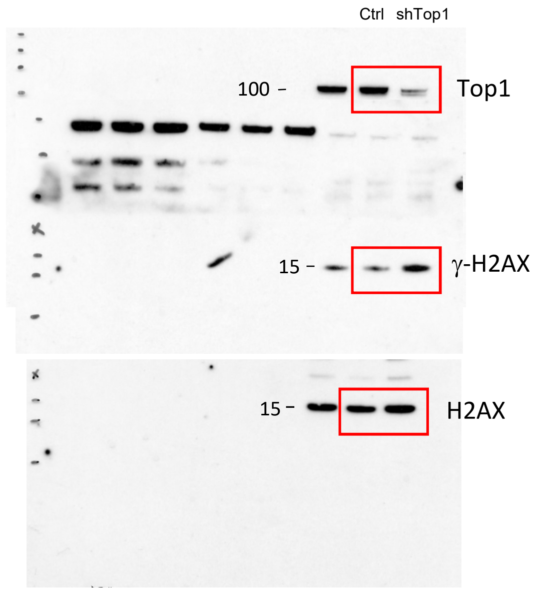

Fig. 4i

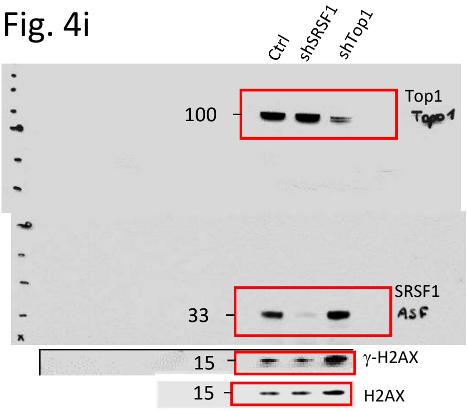

Sup Fig. 2c

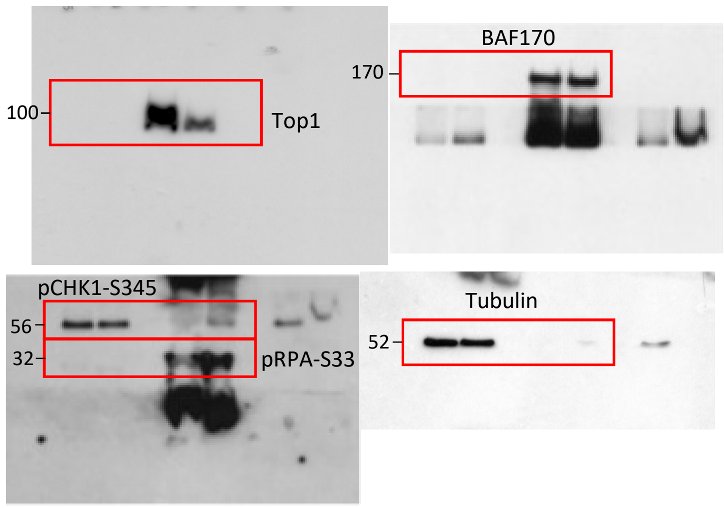

Sup Fig. 4a

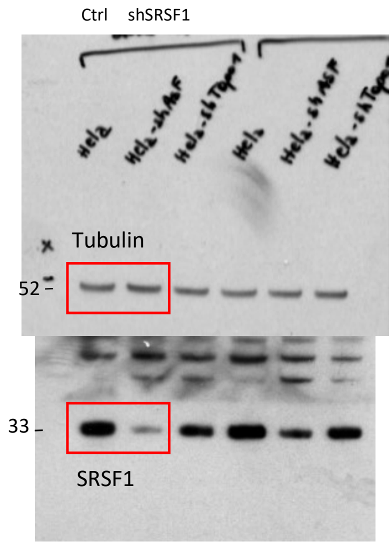

Supplementary Figure 8. Sequential gating strategy for flow cytometry experiments

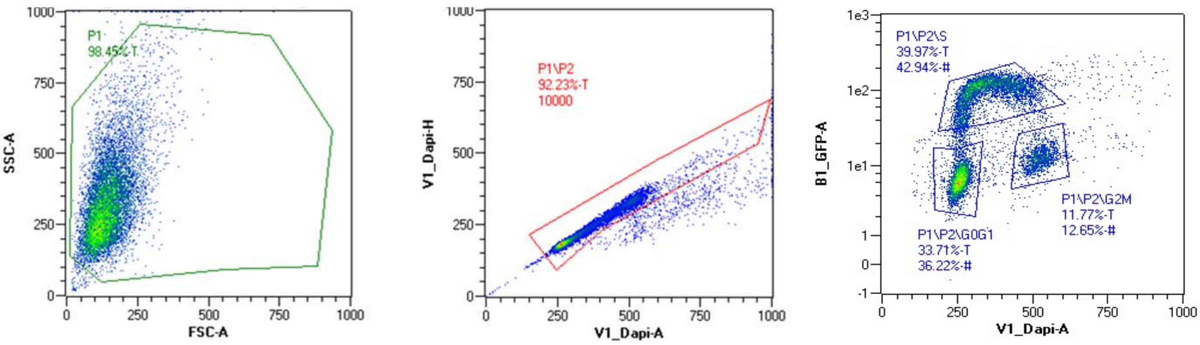

1. Gating of total cell population according to FSC/SSC

2. Gating on DNA content

3. Quantification of EdU intensity on gated cells

Supplementary Table 1: Reagents

Antibodies		
Mouse anti-BrdU clone B44	BD Biosciences	Cat #347580
Rat anti-BrdU clone BU1/75	Eurobio Abcys	Cat #ABC117-7513
Mouse anti-ssDNA	Millipore	Cat #MAB3868
Rabbit anti-pCHK1 (S345)	Cell Signaling Technology	Cat #2348
Mouse anti-γ-H2AX (S139) for WB	Millipore	Cat #05-636
Rabbit anti-γ-H2AX (S139) for ChIP	ABGENT	Cat #AJ1351a
Mouse anti-RNA:DNA hybrid S9.6 hybridoma	ATCC	Cat #HB8730
Rabbit anti-p-RPA (S33)	Bethyl	Cat #A300246A
Rabbit anti-p-RPA (S4/S8)	Bethyl	Cat #A300245A
Rabbit anti RNase H1	Santa Cruz	Cat #sc-30319
Rabbit anti-Actin	Sigma-Aldrich	Cat #A4700
Rat anti-Tubulin	Abcam	Cat #ab6161
Rabbit anti-Top1	Abcam	Cat #ab3825

Chemicals, Peptide, and Recombinant Proteins		
5-iodo-2'-deoxyuridine (IdU)	MP Biomedicals	Cat #2100357
5-chloro-2'-deoxyuridin (CldU)	MP Biomedicals	Cat #2105478
Poly-D-Lysine	Sigma-Aldrich	Cat #P4707
(Hexadimethrine bromide (Polybrene)	Sigma-Aldrich	Cat #H9268
Deoxycycline	Sigma-Aldrich	Cat #D9891

Agarose protein A/G beads	Pierce	Cat #20241
Sepharose protein A beads	Sigma-Aldrich	Cat #P2545
Sepharose protein G beads	Sigma-Aldrich	Cat #P3296
Phusion® High-Fidelity DNA Polymerase	NEB	Cat #M0530S/L

Critical Commercial Assays

Ipure	Diagenode	Cat#C03010012
TruSeq ChIP Library Preparation Kit	Illumina	Cat#IP-202-1024
ThruPLEX® DNA-seq Kit	Rubicon Genomics	R400407
jetPEI DNA Transfection Reagent	Polyplus-transfection	101-10N
TruSeq Stranded mRNA Library Prep Kit	Illumina	N/A
Bioanalyzer 2100 HS DNA Kit	Agilent	Cat#5067-4626
Qubit dsDNA HS Assay Kit	Life Technologies	Cat#Q32851/Q32854
HiSeq Rapid Duo cBot Sample Loading Kit	Illumina	Cat#CT-403-2001

Experimental Models: Cell Lines

Human: HEK293T	ATCC	CRL-3216
Human: HeLa	ATCC	CCL2

Plasmids

pLVX-Tet-on	CLONTECH Laboratories	Cat#632162
TRIPZ-shTop1	Dharmacon	RHS4696-99707195
RNase H1-EGFP	Pommier laboratory	N/A
pEGFP-N1	CLONTECH Laboratories	Cat#6085-1

Supplementary Table 2: Oligonucleotides

MYADM Forward (RNA:DNA positive): CGTAGGTGCCCTAGTTGGGAG	Ginno et al. 2012	N/A
MYADM Reverse (RNA:DNA positive): TCCATTCTCATTCCCAAACC	Ginno et al. 2012	N/A
RPL13A Forward (RNA:DNA positive): AATGTGGCATTTCCTTCTCG	Ginno et al. 2012	N/A
RPL13A Reverse (RNA:DNA positive): CCAATTCGGCCAAGACTCTA	Ginno et al. 2012	N/A
SNRPN Forward (RNA:DNA negative): GCCAAATGAGTGAGGATGGT	Ginno et al. 2012	N/A
SNRPN Reverse (RNA:DNA negative): TCCTCTCTGCCTGACTCCAT	Ginno et al. 2012	N/A
EGR1 downstream Forward (RNA:DNA negative): GAACGTTCAGCCTCGTTCTC	Ginno et al. 2012	N/A
EGR1 downstream Reverse (RNA:DNA negative): GGAAGGTGGAAGGAAACACA	Ginno et al. 2012	N/A
Chr 22 Forward (γ-H2AX negative): CCCATCTCAACCTCCACACT	Iacovoni et al. 2010	N/A
Chr22 Reverse (γ-H2AX negative) : CTTGTCCAGATTCGCTGTGA	Iacovoni et al. 2010	N/A
Chr1 Forward (γ-H2AX positive): TTCCTGCAGCCTCATTTTCT	Iacovoni et al. 2010	N/A
RUNDC3A Forward: ACA CCT GCC ACC TTT CTT CAA	This study	N/A
RUNDC3A Reverse: CGC CAA GGT CAC CAG AAG	This study	N/A
EHBP1L1 Forward: GCC GCC GTA TTT ATT TGT CAC	This study	N/A
EHBP1L1 Reverse: GTC TCG TCC AAC TTT ATT GCT C	This study	N/A
LGALS2 Forward: TGA CCT CAC CTT GAC CTC TGA	This study	N/A
LGALS2 Reverse: AGC TGA ACC TGC ATT TCA ACC	This study	N/A
TUBB Forward: TGG TCC CTA AGC CTC CAG AAA C	This study	N/A
TUBB Reverse: AGG GGG GAA GCA ACA GCA AAA G	This study	N/A
SLC35B2 Forward: AAG TCT TGC CCT AGC TGT GCT	This study	N/A
SLC35B2 Reverse: GCC TAC ACC GCT TGT GCT TTT	This study	N/A
RPL12 Forward: CCG CCA TCC TCA TGA CAT CAT C	This study	N/A

RPL12 Reverse:<br>CAC CCC ACC AGT AAC CAT TTC C	This study	N/A
GYS1 Forward:<br>GCT TAT TCC CAA CTG CTC TGC C	This study	N/A
GYS1 Reverse:<br>ACA CAC TCC AAT CAC ACC CCT C	This study	N/A
HTR1E Forward:<br>TGA GCA TCT ACA CCG TGT CC	This study	N/A
HTR1E Reverse:<br>TCA CAT AAC CGA GCC ACG TC	This study	N/A
NR134609 Forward:<br>TCA GAC TCT TTG TGC TCT CC	This study	N/A
NR134609 Reverse:<br>GCT TGG GTT CCT CAT TTG ATT	This study	N/A
HIST2H4B Forward:<br>CTC CAG CTT TGC ACG TTT C	This study	N/A
HIST2H4B Reverse:<br>GCA GTC AAG TGT CCA ACT CC	This study	N/A
i-BLESS adapter P5: P-<br>GATCGGAAGAGCGTCGTGTAGGGAAAGAGTGTU<br>UU[btnT<br>]UUUACACTCTTTCCCTACACGACGCTCTTCCGAT<br>CT	This study	N/A
i-BLESS adapter P7: P-<br>GATCGGAAGAGCACACGTCTGAACTCCAGTCAC<br>UUUUUUUGTGACTGGAGTTCAGACGTGTGCTCTT<br>CCGATCT	This study	N/A
i-BLESS PCR primer:<br>AATGATACGGCGACCACCGAGATCTACACTCTTT<br>CCCTACACGAC	This study	N/A
i-BLESS index primer:<br>CAAGCAGAAGACGGCATACGAGAT[INDEX]GTG<br>ACTGGAGTTCAGACGT	This study	N/A
