# Supplementary material for: Topoisomerase 1 prevents replication stress at R-loop-enriched transcription termination sites
Source: Nat Commun. 2020 Aug 7;11:3940. doi: 10.1038/s41467-020-17858-2 (PMC7414224; doi:10.1038/s41467-020-17858-2)
Supplement: Supplementary file 3 — Reporting summary [file 41467_2020_17858_MOESM3_ESM.pdf]

nature research

Corresponding author(s): Philippe Pasero; Yea-Lih Lin; Chunlong Chen

Last updated by author(s): Jun 25, 2020

Reporting Summary

Nature Research wishes to improve the reproducibility of the work that we publish. This form provides structure for consistency and transparency in reporting. For further information on Nature Research policies, see our Editorial Policies and the Editorial Policy Checklist.

Statistics

For all statistical analyses, confirm that the following items are present in the figure legend, table legend, main text, or Methods section.

n/a	Confirmed	
☐	☒	The exact sample size (*n*) for each experimental group/condition, given as a discrete number and unit of measurement
☒	☐	A statement on whether measurements were taken from distinct samples or whether the same sample was measured repeatedly
☐	☒	The statistical test(s) used AND whether they are one- or two-sided<br>*Only common tests should be described solely by name; describe more complex techniques in the Methods section.*
☒	☐	A description of all covariates tested
☒	☐	A description of any assumptions or corrections, such as tests of normality and adjustment for multiple comparisons
☐	☒	A full description of the statistical parameters including central tendency (e.g. means) or other basic estimates (e.g. regression coefficient) AND variation (e.g. standard deviation) or associated estimates of uncertainty (e.g. confidence intervals)
☐	☒	For null hypothesis testing, the test statistic (e.g. *F*, *t*, *r*) with confidence intervals, effect sizes, degrees of freedom and *P* value noted<br>*Give P values as exact values whenever suitable.*
☒	☐	For Bayesian analysis, information on the choice of priors and Markov chain Monte Carlo settings
☒	☐	For hierarchical and complex designs, identification of the appropriate level for tests and full reporting of outcomes
☒	☐	Estimates of effect sizes (e.g. Cohen's *d*, Pearson's *r*), indicating how they were calculated

Our web collection on statistics for biologists contains articles on many of the points above.

Software and code

Policy information about availability of computer code

Data collection	For NGS-based experiments, quality and quantity of libraries were assessed on 2100 Bioanalyzer using HS DNA Kit (Agilent), and on Qubit 2.0 Fluorometer using Qubit dsDNA HS Assay Kit (Life Technologies). The libraries were sequenced (1x61 bp) on Illumina HiSeq2500 platform, according to the modified experimental and software protocols for generation of high-quality data for low-diversity samples (Mitra, 2015)
Data analysis	ChIP-seq and DRIP-seq data were aligned to Human genome reference (hg19 assembly) with Bowtie2 and RNA-seq using STAR v2.7. Mapping quality was assessed with SAMtools v1.1 and in-house Python scripts. The quality of sequencing data was assessed with FastQC (http://www.bioinformatics.babraham.ac.uk/projects/fastqc). Peak-calling was done using MACS2. Reproducible peaks from replicates were then selected using the Irreproducible Discovery Rate (IDR) method from ENCODE Project. Intersection of transcript annotations with R-loop was done using BEDTools v2.28. DeepTools2 was used to compute and draw enrichment heat maps and profiles on positions of interest. Further analyses were done in R with Bioconductor packages and ggplot2 for graphic representation. The code generated during the current study is available from the corresponding authors. The DNA fibers were measured by MetaMorph Microscopy Automation and Image Analysis Software v7.8 (Molecular Devices) and statistical analysis was performed with GraphPad Prism 8 (GraphPad Software). Flow cytometry data were analyzed using FlowJo 10 (LLC).

For manuscripts utilizing custom algorithms or software that are central to the research but not yet described in published literature, software must be made available to editors and reviewers. We strongly encourage code deposition in a community repository (e.g. GitHub). See the Nature Research guidelines for submitting code & software for further information.

Data

Policy information about availability of data

All manuscripts must include a data availability statement. This statement should provide the following information, where applicable:

- Accession codes, unique identifiers, or web links for publicly available datasets
- A list of figures that have associated raw data
- A description of any restrictions on data availability

The datasets generated during and/or analyzed during the current study are available from the corresponding author on reasonable request. The NGS datasets generated and analyzed during the current study are available in the GEO repository, accession number: GSE108172.

Field-specific reporting

Please select the one below that is the best fit for your research. If you are not sure, read the appropriate sections before making your selection.

[x] Life sciences [ ] Behavioural & social sciences [ ] Ecological, evolutionary & environmental sciences

For a reference copy of the document with all sections, see nature.com/documents/nr-reporting-summary-flat.pdf

Life sciences study design

All studies must disclose on these points even when the disclosure is negative.

Sample size	Sample size was determined according to standards in the field. For DNA fiber spreading, at least 150 fibers were measured as recommended (Bianco, 2012). For comet assay, the tail length of 50-100 cells were measured as described (Tuduri, 2009). Quantification of Mean Fluorescence Intensity (MFI) was performed on more than 400 cells.
Data exclusions	No data were excluded.
Replication	All genomic datasets were reproduced at least twice with the same method, except for DSB mapping that was performed once with the original BLESS assay and once with the improved i-BLESS protocol. All attempts at replication were successful, unless when immunoprecipitation experiments were not efficient enough due to variability in the quality of antibodies.
Randomization	Randomization was not relevant to our study. All cell lines or biological samples were analysed or treated in the same manner.
Blinding	Blinding was used for DNA fiber analysis (Bianco, 2012). Blinding was not relevant to other experiments in this study.

Reporting for specific materials, systems and methods

We require information from authors about some types of materials, experimental systems and methods used in many studies. Here, indicate whether each material, system or method listed is relevant to your study. If you are not sure if a list item applies to your research, read the appropriate section before selecting a response.

Materials & experimental systems

n/a	Involved in the study	
[ ]	[x]	Antibodies
[ ]	[x]	Eukaryotic cell lines
[x]	[ ]	Palaeontology and archaeology
[x]	[ ]	Animals and other organisms
[x]	[ ]	Human research participants
[x]	[ ]	Clinical data
[x]	[ ]	Dual use research of concern

Methods

n/a	Involved in the study	
[ ]	[x]	ChIP-seq
[ ]	[x]	Flow cytometry
[x]	[ ]	MRI-based neuroimaging

Antibodies

Antibodies used

Antibodies	Sources	Cat no.
Mouse anti-BrdU clone B44	BD Biosciences	Cat #347580
Rat anti-BrdU clone BU1/75	Eurobio Abcys	Cat #ABC117-7513
Mouse anti-ssDNA	Millipore	Cat #MAB3868
Rabbit anti-pCHK1 (S345)	Cell Signaling Technology	Cat #2348
Mouse anti--H2AX (S139) for WB	Millipore	Cat #05-636
Rabbit anti--H2AX (S139) for ChIP	ABGENT	Cat #AJ1351a
Mouse anti-RNA:DNA hybrid S9.6hybridoma	ATCC	Cat #HB8730

Rabbit anti-p-RPA (S33)	Bethyl	Cat #A300246A
Rabbit anti-Nucleolin	Abcam	Cat #ab22758
Rabbit anti RNase H1	Santa Cruz	Cat #sc-30319
Rabbit anti-Actin	Sigma-Aldrich	Cat #A4700
Rat anti-Tubulin	Abcam	Cat #ab6161
Rabbit anti-Top1	Abcam	Cat #ab3825

Validation

All the antibodies applied in the study are commercially available and were validated by the respective companies.

Eukaryotic cell lines

Policy information about cell lines

Cell line source(s)

Human embryonic kidney (HEK) HEK293T cells (ATCC CRL-3216)
HeLa cells (ATCC CCL-2) were purchased from ATCC. U2OS-DIvAcells were obtained from Legube Lab (Iacovoni et al. 2010).

Authentication

None of the cell lines have been authenticated by Standards for Cell Line Authentification (Almeida et al. 2016). However, HEK293T and HeLa cells have been purchased from ATCC and authenticated by this organization with certificates. DIvA cells were from Legube Lab (Iacovoni et al. 2010).

Mycoplasma contamination

All the cell lines are mycoplasma-free. They have been tested for mycoplasma contamination regularly using MycoAlert Mycoplasma Detection Kit (LONZA). All cell lines tested were mycoplasma negative.

Commonly misidentified lines
(See ICLAC register)

No commonly misdefined cell lines were used in the study.

ChIP-seq

Data deposition

☒ Confirm that both raw and final processed data have been deposited in a public database such as GEO.

☒ Confirm that you have deposited or provided access to graph files (e.g. BED files) for the called peaks.

Data access links
May remain private before publication.

The NGS datasets generated during the current study are available in the GEO repository, accession number: GSE108172.

Files in database submission

For control, shTop1 and shSRSF1 cells, files in database submission include DRIP-seq (duplicates), ChIP-seq (gamma-H2AX, pRPA32 S33; duplicates), BLESS, i-BLESS, RNA-seq.
For U2OS DIvA cells, files in database include ChIP-seq of gamma-H2AX before and after Tam induction.

Genome browser session
(e.g. UCSC)

No longer applicable

Methodology

Replicates

Biological replicates were performed for all experiments

Sequencing depth

Sequencing depth ranges from 56 million to 432 million reads per sample, single-reads.

Antibodies

Rabbit anti-p-RPA (S33) Bethyl Cat #A300246A
Rabbit anti-gamma-H2AX (S139) for ChIP ABGENT Cat #AJ1351a
Mouse anti-RNA:DNA hybrid S9.6 hybridoma ATCC Cat #HB8730

Peak calling parameters

Peak-calling for DRIP seq data was done using MACS285 with a q-value of 0.05 and keeping up to five replicates. Reproducible peaks from replicates were then selected using the Irreproducible Discovery Rate (IDR) method from ENCODE Project86, with a cutoff value of 0.05.

Data quality

The quality of sequencing data was assessed with FastQC (http://www.bioinformatics.babraham.ac.uk/projects/fastqc) and in-house PERL and Python scripts.

Software

Peak-calling was done using MACS2. Intersection of transcripts annotation (RefSeq, hg19) with R-loop signal was done using BEDTools. The analyses of replication fork directionality and replication initiation zones used the published OK-seq data from HeLa cells. DeepTools2 was used to compute and draw enrichment heat maps and profiles on positions of interest (peaks, TSS, TTS). Further analyses were done in R (http://www.R-project.org), with Bioconductor packages and ggplot2 for graphic representation.

Flow Cytometry

Plots

Confirm that:

- [x] The axis labels state the marker and fluorochrome used (e.g. CD4-FITC).
- [x] The axis scales are clearly visible. Include numbers along axes only for bottom left plot of group (a 'group' is an analysis of identical markers).
- [x] All plots are contour plots with outliers or pseudocolor plots.
- [x] A numerical value for number of cells or percentage (with statistics) is provided.

Methodology

Sample preparation	Cells were labeled with EdU for 15 minutes, then fixed with 2% PFA before proceeding to Click reaction and FACS analyses.
Instrument	MACSQuant analyser 10 Flow Cytometer
Software	FlowJo v10 (LLC)
Cell population abundance	10,000 Cells with appropriate FSC/SSC and the characteristics of single cells were collected and analyzed.
Gating strategy	Cells with appropriate FSC/SSC were gated first, the single cell population was further gated for the analyses of cell cycle distribution.

- [x] Tick this box to confirm that a figure exemplifying the gating strategy is provided in the Supplementary Information.
