# Supplementary material for: Topoisomerase 1 prevents replication stress at R-loop-enriched transcription termination sites
Source: Nat Commun. 2020 Aug 7;11:3940. doi: 10.1038/s41467-020-17858-2 (PMC7414224; doi:10.1038/s41467-020-17858-2)
Supplement: Supplementary file 2 — Peer review [file 41467_2020_17858_MOESM2_ESM.pdf]

Reviewers' Comments:

Reviewer #1:
Remarks to the Author:

In this manuscript, Promonet, Padioleau, Liu et al use ChIP-seq to map the distribution of R-loops, phospho-RPA (pRPA) and γ-H2AX around transcribed genes in HeLa cells, both under normal conditions and during mild depletion of Top1. Interestingly, the authors find that although R-loops are enriched at both the transcription start site (TSS) and transcription termination site (TTS), pRPA is specifically enriched at TTS. Additionally, the enrichment of R-loops and pRPA is substantially elevated at convergent genes that are actively transcribed. When Top1 is depleted, γ-H2AX is enriched around sites bound by pRPA, suggesting that Top1 acts to prevent the formation of double-strand breaks at these sites. The authors extend this observation by performing BLESS to analyze the distribution of DSBs: DSBs are enriched at TSS, but are dramatically increased at TTS upon Top1 depletion in about a quarter of genes. The authors synthesize these data into a model whereby replication forks encountering RNA polymerase head-on at TTS stall and activate ATR, thereby preventing further replication into the gene. When Top1 is depleted, the resulting topological stress leads to fork collapse and DSBs

Overall I think this is a nicely executed study that advances the field enough to warrant publication in Nature Communications with few revisions. The authors make careful and interesting observations, which come together into a coherent model that is not only consistent with recent literature, but also extends the conceptual framework of how TRCs and R-loops impact DNA replication. I do not think additional experimental work is required before publication, and my only real criticism is that the manuscript feels a bit too condensed. It’s hard to intuit exactly how some of the analyses were done, even after thorough and repeated readings (e.g. what is the basis of the five quintiles in figure 3b? How was the clustering done for Figure 4?). The authors should ensure that the overall workflow is a bit easier to follow.

Reviewer #2:
Remarks to the Author:
In this manuscript, Pasero and colleagues address the question of whether all R-loops are created equal in their ability to cause DNA damage, using Top1 knockdown as a tool to enhance the formation of R-loops at certain genomic sites. Consistent with previous reports, their data suggest that R-loops form predominantly at transcription start sites (TSS) and transcription termination sites (TTS), and that Top1 knockdown enhances R-loop formation specifically at TTS. Using pS33 RPA, a marker of ATR activation, and gammaH2AX, a marker of ATR, ATM and DNAPK activation, they then go on to show that the head-on convergence of a replication fork with a TTS leads to replication stress, marked by pRPA. In the absence of Top1, problems resulting from such convergence are enhanced and forks seem to collapse, leading to DSB formation. This is supported by the increased phosphorylation of H2AX and increases in DSB formation at TTS sites where fork convergence occurs.

This is an exciting manuscript that sheds new insight into a very important question in R-loop field about the similarities and differences in R-loops. It also helps address the question of how R-loops mechanistically cause damage. Using a variety of genomic approaches, the authors demonstrate that just a subset of R-loops are causing problems for the cell -- those where forks converge head on with transcription termination sites. They also go on to restate the model as one in which transient fork pausing occurs at sites of transcription and R-loop formation, to ultimately help suppress a collision. Generally I think this is a very well done study and the data are interpreted carefully. It is quite appropriate for Nat Comm and should be of broad interest. However, there are some relatively minor issues that should be addressed before publication, and I have a few suggestions that will strengthen the work.

Specific Comments:
The authors should in some way validate the formation of DSBs in their system. Both H2AXp and RPAp (on this site) can be replication stress markers and neither is specific to DSBs. It would be helpful to show by comet assay or another approach (e.g. with a more DSB specific marker) that breaks are forming under the shTOP1 conditions used here.

A related/overlapping point is whether the authors can show that the increased H2AX resulting from Top1 knockdown is really due to break formation and ATM activation at TTSs. Does an ATM inhibitor and not ATR inhibitor reduce this signal; or is a more break-specific marker induced at some of these sites?

A few sites of RPAp and hybrid formation at the TTS of several converging genes with and without Top1 should be validated using qPCR approaches. A related point is that the authors should validate that DRIP signal is up using qPCR at the TTS (and not over the whole gene) upon Top1 knockdown.

The authors should include a scatter plot (or some other genome-wide analysis) to compare R-loops after Top1 knockdown and before. Do all sites go up or are some down as well?

Related to the last point, my understanding is that Manzo et al (2019) didn't observe the same differential effects at the TSS and TES upon TOP1 depletion if I understand that work correctly. Given that one of the authors here is an author on both papers they must be aware of this and should address the potential differences.

There is little explanation or speculation of why Top1 might be particularly important in preventing breaks at the TES. I think it would strengthen the manuscript to come back to this in the discussion in a little more detail.
Please compare RPA sites with and without Top1 knockdown – it's not clear if the old sites are the same as the new one or if the sites are new after knockdown.

Figure 2a and d – is this all with Top1 knockdown? How does it compare to signal in control cells. Figure 2d should include this comparison and a screen shot (for 2a) comparing results with and without Top1 would be helpful.

It's unclear how the clustering in Figure 4 was done - there appear to be a number of positive regions in their "negative" cluster 2, and a number of negative regions in their "positive" cluster 1 in the control samples, so it is assumed that they only took into account the siTOP1 condition when doing this clustering. It seems surprising to perform clustering when you only end up with 2 categories (vs multiple dimensions). Why not just rank them from most positive to least positive, and set some sort of threshold? If these clusters represent distinct categories of genes (i.e. induced in TOP1 vs decreased), the names of the clusters should reflect this or at least be more informative.

The significance of some of the H2AX data may be overstated or overinterpretated. To this reviewer it appears high in general over genic regions when Top1 is knocked down. Additionally, in the metaplots shown in 3B, the signal doesn't seem to resolve to the site of the TTS very well. It might help to show, for example, that there is no accumulation around the closest TSS to each observed TTS. Alternatively, the authors could potentially aggregate signal over a broader window (say 200kb on each side)? Related to this, in figure 4, why do the levels of pH2AX seem to be so much higher in both categories of genes in the shTOP1 condition. These data suggest that there is nothing particularly special about the TTS in terms of pH2AX. Based on these concerns, I think it the authors make too much about the H2AX data and may want to soften their language, particularly since the BLESS data shows a much higher-resolution and more convincing picture of damage at these sites.

Are the places where DSBs are increased by iBLESS the same places where forks converge on TTS in a close range?

Could the authors provide error bands on the metaplots (e.g. Figure 1g, 2d, 4b).

Minor points:
Figure S1b – are these samples all with shTop1? Or control?
Is the data in Figure S2a the same as that in 2b, top panel

Reviewers' comments:

Reviewer #1 (Remarks to the Author):
In this manuscript, Promonet, Padioleau, Liu et al use ChIP-seq to map the distribution of R-loops, phospho-RPA (pRPA) and γ-H2AX around transcribed genes in HeLa cells, both under normal conditions and during mild depletion of Top1. Interestingly, the authors find that although R-loops are enriched at both the transcription start site (TSS) and transcription termination site (TTS), pRPA is specifically enriched at TTS. Additionally, the enrichment of R-loops and pRPA is substantially elevated at convergent genes that are actively transcribed. When Top1 is depleted, γ-H2AX is enriched around sites bound by pRPA, suggesting that Top1 acts to prevent the formation of double-strand breaks at these sites. The authors extend this observation by performing BLESS to analyze the distribution of DSBs: DSBs are enriched at TSS, but are dramatically increased at TTS upon Top1 depletion in about a quarter of genes. The authors synthesize these data into a model whereby replication forks encountering RNA polymerase head-on at TTS stall and activate ATR, thereby preventing further replication into the gene. When Top1 is depleted, the resulting topological stress leads to fork collapse and DSBs

Overall I think this is a nicely executed study that advances the field enough to warrant publication in Nature Communications with few revisions. The authors make careful and interesting observations, which come together into a coherent model that is not only consistent with recent literature, but also extends the conceptual framework of how TRCs and R-loops impact DNA replication. I do not think additional experimental work is required before publication, and my only real criticism is that the manuscript feels a bit too condensed. It's hard to intuit exactly how some of the analyses were done, even after thorough and repeated readings (e.g. what is the basis of the five quintiles in figure 3b? How was the clustering done for Figure 4?). The authors should ensure that the overall workflow is a bit easier to follow.

We are grateful to this Reviewer for his/her enthusiastic assessment of our work. We agree that the manuscript was too condensed and we have now developed several sections to address this issue. These changes are marked in red in the manuscript. In particular, we now explain how the five quintiles in **Fig. 3b** (now **3d**) were generated. We sorted the 35251 annotated genes according to their mRNA level (RPKM) and divided this list in five quintiles of 7050 genes. We also better explain how i-BLESS data were analyzed and we now include a novel analysis suggested by Reviewer #2. We hope that this Reviewer will agree on the fact that we have greatly improved our manuscript.

Reviewer #2 (Remarks to the Author):
In this manuscript, Pasero and colleagues address the question of whether all R-loops are created equal in their ability to cause DNA damage, using Top1 knockdown as a tool to enhance the formation of R-loops at certain genomic sites. Consistent with previous reports, their data suggest that R-loops form predominantly at transcription start sites (TSS) and transcription termination sites (TTS), and that Top1 knockdown enhances R-loop formation specifically at TTS. Using pS33 RPA, a marker of ATR activation, and gammaH2AX, a marker of ATR, ATM and DNAPK activation, they then go on to show that the head-on convergence of a replication fork with a TTS leads to replication stress, marked by pRPA. In the absence of Top1, problems resulting from such convergence are enhanced and forks seem to collapse, leading to DSB formation. This is supported by the increased phosphorylation of H2AX and increases in DSB formation at TTS sites where fork convergence occurs.

This is an exciting manuscript that sheds new insight into a very important question in R-loop field about the similarities and differences in R-loops. It also helps address the question of how R-loops mechanistically cause damage. Using a variety of genomic approaches, the authors demonstrate that just a subset of R-loops are causing problems for the cell -- those where forks converge head on with transcription termination sites. They also go on to restate the model as one in which transient fork pausing occurs at sites of transcription and R-loop formation, to ultimately help suppress a collision. Generally I think this is a very well done study and the data are interpreted carefully. It is quite appropriate for Nat Comm and should be of broad interest. However, there are some relatively minor issues that should be addressed before publication, and I have a few suggestions that will strengthen the work.

We thank this reviewer for his/her constructive comments and for helping us improve our manuscript.

Specific Comments:

The authors should in some way validate the formation of DSBs in their system. Both H2AXp and RPAp (on this site) can be replication stress markers and neither is specific to DSBs. It would be helpful to show by comet assay or another approach (e.g. with a more DSB specific marker) that breaks are forming under the shTOP1 conditions used here.

We have performed a comet assay and the immunodetection of the DSB marker p-RPA32 (S4/S8). Both assays confirmed an increase of DNA breaks in shTop1 cells relative to control HeLa cells. These results are shown in **Fig. 3b** and **3c**.

A related/overlapping point is whether the authors can show that the increased H2AX resulting from Top1 knockdown is really due to break formation and ATM activation at TTSs. Does an ATM inhibitor and not ATR inhibitor reduce this signal; or is a more break-specific marker induced at some of these sites?

As mentioned above, we have confirmed that DSBs form more frequently in shTop1 cells, which would explain why they accumulate more γ-H2AX than control cells. Unfortunately, we have been unable to determine whether this phosphorylation is due to ATR, ATM or DNA-PK, as the treatment with kinase inhibitors was sufficient by itself to induce pRPA32 S4/S8 foci (see examples below for VE-822 and ETP46464 inhibitors; Fig. 1 for reviewers).

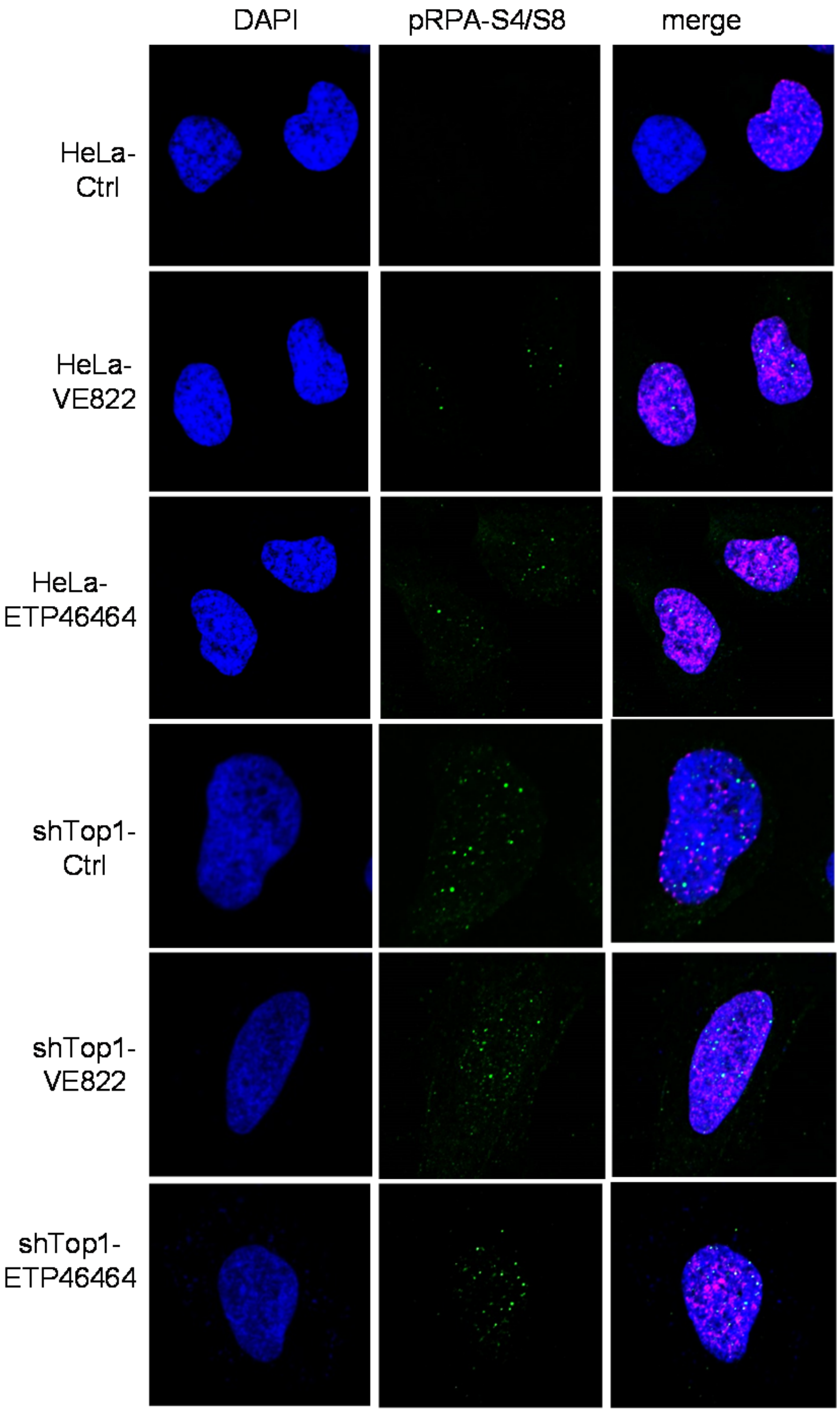

Figure 1 for reviewers: Effect of VE822 and ETP46464 on p-RPA32 (S4/S8) foci. Control and shTop1 HeLa cells were treated with doxycycline for 72 hours. Cells were labeled with EdU for 15 min, washed and then treated with 10 µM VE-822 or 10 µM ETP-46464 for 1 hour before detection of p-RPA32 (S4/S8) foci. Blue: DAPI; green: p-RPA32 (S4/48); red: EdU.

A few sites of RPAp and hybrid formation at the TTS of several converging genes with and without Top1 should be validated using qPCR approaches. A related point is that the authors should validate that DRIP signal is up using qPCR at the TTS (and not over the whole gene) upon Top1 knockdown.

We have now validated the enrichment of RNA:DNA hybrids (**Fig. 1g**) and p-RPA32 S33 (**Fig. S2d**) at the TTS of several genes by qPCR in control and shTop1 cells.

The authors should include a scatter plot (or some other genome-wide analysis) to compare R-loops after Top1 knockdown and before. Do all sites go up or are some down as well?

As requested by this Reviewer, we have now included a scatter plot to compare the intensity of R-loops before and after Top1 knockdown (new **Supplementary Fig. 1j**). This analysis shows that genes that are specifically enriched in R-loops in shTop1 cells have a relatively weak DRIP signal intensity relative to other genes. This is consistent with the fact that these genes show also a lower level of expression (new **Supplementary Fig. 1i**).

Related to the last point, my understanding is that Manzo et al (2019) didn't observe the same differential effects at the TSS and TES upon TOP1 depletion if I understand that work correctly. Given that one of the authors here is an author on both papers they must be aware of this and should address the potential differences.

This difference is due to the extent of Top1 depletion. In Manzo et al. (2019), Top1 was strongly depleted, to the point that cells stopped proliferating. Here, the reduction of Top1 levels was sufficient to slow down fork progression, but did not affect cell cycle progression (**Fig. 1b**). Cells are therefore in different physiological conditions, which would explain the difference observed in the two studies in terms of R-loop distribution. This is now better explained in the manuscript.

There is little explanation or speculation of why Top1 might be particularly important in preventing breaks at the TES. I think it would strengthen the manuscript to come back to this in the discussion in a little more detail.

We thank this Referee for pointing this out. The mechanism by which Top1 prevents breaks was difficult to address since Top1 prevents both the accumulation of topological stress and the formation of R-loops. To separate the relative contribution of R-loops and topological stress in break formation, we have analyzed the distribution of R-loops, p-RPA and γ-H2AX in cells depleted for SRSF1, a splicing factor preventing the formation of R-loops without affecting DNA topology. In new **Fig. 4** and **Supplementary Fig. 4**, we show that although the depletion of SRSF1 increases the formation of R-loops at TSS and TTS, it does not induce the accumulation of γ-H2AX to the same extent as in shTop1 cells. These data support the view that R-loops are necessary but not sufficient to induce DSBs and that Top1 prevents breaks by relaxing supercoiled DNA when replication and transcription converge at TTS.

Please compare RPA sites with and without Top1 knockdown – it's not clear if the old sites are the same as the new one or if the sites are new after knockdown.

We now indicate in the manuscript (p. 6) that 97% of the p-RPA S33 sites observed in shTop1 cells were also detected in control cells. This indicates that the same sites accumulate p-RPA after Top1

knockdown.

Figure 2a and d – is this all with Top1 knockdown? How does it compare to signal in control cells. Figure 2d should include this comparison and a screen shot (for 2a) comparing results with and without Top1 would be helpful.

Fig. 2a (MED15 locus), **2d** (metaplot) and **2e** (KDM1A-LUZP1 locus) correspond to control cells. The corresponding data for shTop1 cells are now shown in **Supplementary Fig. 2a, 2e and 2f**.

It's unclear how the clustering in Figure 4 was done - there appear to be a number of positive regions in their "negative" cluster 2, and a number of negative regions in their "positive" cluster 1 in the control samples, so it is assumed that they only took into account the siTOP1 condition when doing this clustering. It seems surprising to perform clustering when you only end up with 2 categories (vs multiple dimensions). Why not just rank them from most positive to least positive, and set some sort of threshold? If these clusters represent distinct categories of genes (i.e. induced in TOP1 vs decreased), the names of the clusters should reflect this or at least be more informative.

As suggested by this reviewer, we have now ranked genes from most positive to least positive i-BLESS signal at TTS in shTop1 cells and have applied a threshold (Top 25%). This new analysis is now shown in a new **Fig. 5**. Since this analysis shows virtual the same results as for the previous clustering approach, we also kept the original clustering analysis in **Supplementary Fig. 5**. We have also analyzed the intensity of i-BLESS signal at TSS using the same parameters as for TTS in **Fig. 5**. This analysis is shown in **Supplementary Fig. 5c** and **5d**.

The significance of some of the H2AX data may be overstated or overinterpretated. To this reviewer it appears high in general over genic regions when Top1 is knocked down. Additionally, in the metaplots shown in 3B, the signal doesn't seem to resolve to the site of the TTS very well. It might help to show, for example, that there is no accumulation around the closest TSS to each observed TTS. Alternatively, the authors could potentially aggregate signal over a broader window (say-H2AX 200kb on each side)?

We agree with this Referee that the analysis of the γ-H2AX signal is complicated by the fact that this signal spreads over large regions, encompassing the TSS of the same gene and several nearby genes. This is now illustrated in a control ChIP-seq experiment using the DIvA system to create DSBs at specific *Asi*SI sites (**Supplementary Fig. 3a**). We have therefore toned down the interpretation of γ-H2AX and put more emphasis on DSB signals.

Related to this, in figure 4, why do the levels of pH2AX seem to be so much higher in both categories of genes in the shTOP1 condition. These data suggest that there is nothing particularly special about the TTS in terms of pH2AX. Based on these concerns, I think it the authors make too much about the H2AX data and may want to soften their language, particularly since the BLESS data shows a much higher-resolution and more convincing picture of damage at these sites.

The high level of γ-H2AX on metaplots reflects the fact that this mark spreads over long distances, as illustrated also in **Fig. 3d** and **Supplementary Fig. 3a**. We kept this panel in the new **Supplementary Fig.**

5b as it shows that regions enriched in i-BLESS signal are also enriched in γ-H2AX, but we are happy to remove it completely if Reviewer #2 finds it confusing.

Are the places where DSBs are increased by iBLESS the same places where forks converge on TTS in a close range?

Yes, i-BLESS and p-RPA signals are enriched in a close range (i.e. few kb around TTS), see **Fig 5b**.

Could the authors provide error bands on the metaplots (e.g. Figure 1g, 2d, 4b).

We now provide error bars (SEM) on all metaplots.

Minor points:
Figure S1b – are these samples all with shTop1? Or control?

These samples are from control cells.

Is the data in Figure S2a the same as that in 2b, top panel

The data in **Supplementary Fig. 2a** (now **Supplementary Fig. 2e**) correspond to shTop1 cells, whereas data in **Fig. 2b** (now **Fig. 2d**) correspond to control cells. This is now indicated in the figures and figure legends.

Reviewers' Comments:

Reviewer #2:
Remarks to the Author:
The authors have done a wonderful job addressing my concerns and I think this is really a beautiful paper - congrats to them.

A few minor points for the final version
- in the abstract, I think the worked "accumulated" instead of "cumulated" is more appropriate.
- figure 4 g, h - labeling the colors of lines and dots on the figure itself would help

Reviewer #2 (Remarks to the Author):

The authors have done a wonderful job addressing my concerns and I think this is really a beautiful paper - congrats to them.

We thank this reviewer for his/her positive comments and for helping us improve the manuscript.

A few minor points for the final version
- in the abstract, I think the worked "accumulated" instead of "cumulated" is more appropriate.

Indeed, this is now corrected.

- figure 4 g, h - labeling the colors of lines and dots on the figure itself would help

We have labeled the colors of lines and dots on figure 4, as requested.
